# Nelfinavir triggers ferroptosis by inducing ER stress mediated downregulation of GPX4/GSH system, upregulation of NRF2/HO-1 axis, and mitochondrial impairment in hepatocellular carcinoma cells

**DOI:** 10.1038/s41420-025-02761-w

**Published:** 2025-10-06

**Authors:** Lei Zhang, Xuejun Wang

**Affiliations:** 1 Division of Biomedical and Translational Sciences, The University of South Dakota Sanford School of Medicine, Vermillion, SD USA; 2 College of Veterinary Medicine, Jilin Agricultural University, Changchun, China

**Keywords:** Macroautophagy, Cancer therapeutic resistance, Lysosomes

## Abstract

Hepatocellular carcinoma (HCC) is one of the most common malignancies with poor prognosis. Novel therapeutic strategies for HCC are urgently needed. Ferroptosis, an iron and reactive oxygen species (ROS) dependent regulated cell death, emerges to efficiently abrogate the growth and proliferation of HCC cells. The identification of new ferroptosis inducing agents should provide potential therapeutics for more effective management of HCC. Here we have identified nelfinavir, a human immunodeficiency virus (HIV) protease inhibitor as a novel ferroptosis inducer in HCC cells, Hepa1-6 and HepG2. Mechanistically, the induction of ferroptosis by nelfinavir required its induction of ER stress; suppression of ER stress remarkably attenuated mitochondrial impairment and superoxide production, the autophagic degradation of GPX4, and increases in the labile iron pool associated with the activation of the nuclear factor erythroid 2-related factor 2 (Nrf2)/heme oxygenase-1 (HO-1) axis in nelfinavir-treated HCC cells. In a mouse model of HCC xenografts, nelfinavir treatment significantly suppressed tumor growth, and this effect was more pronounced when nelfinavir and sorafenib were administered together. Collectively, we demonstrate that nelfinavir can induce ferroptosis in an ER stress dependent manner, thereby identifying a new inducer of ferroptosis that can potentially be repurposed to treat HCC.

## Introduction

Liver malignancies are one of the most common solid tumors and a leading cause of cancer-related death worldwide. Hepatocellular carcinoma (HCC) is the predominant form of liver cancer, representing 75–85% of total cases [1]. Late diagnosis, tumor recurrence, therapeutic unresponsiveness and liver dysfunction are the main reasons that hold back HCC patients’ survival rate [1, 2]. It is urgent to develop novel therapeutic approaches for this devastating disease. In the clinic, integration of novel targeted therapeutic agents into standard therapy is critical to increasing patient survival and improving their overall quality of life [3]. Molecular targeted therapy provides a good choice for HCC treatment by targeting specific cell signaling pathways, oncogenes and cytokines. Currently, multi-kinase inhibitor sorafenib is the only FDA-approved first-line treatment for advanced HCC [4]. The success of sorafenib indicates the great opportunity to further explore novel drugs for molecular therapy of HCC.

Ferroptosis is an iron-dependent form of regulated necrosis, executed by oxidized phospholipids containing polyunsaturated fatty acids (PUFA) mediated membrane damage [5]. Emerging studies have revealed the therapeutic potential of ferroptosis in cancer treatment. The anti-tumoral effects of several conventional cancer therapies are partially mediate by ferroptosis, including radiation therapy [6], targeted therapy [7], chemotherapy [8], and immunotherapy [9]. Meanwhile, ferroptosis inducers also potentiate the efficacy of conventional therapies [8]. In addition, growing evidence suggests that ferroptosis could overcome resistance to targeted therapy [10]. It has been reported that ferroptosis could selectively target cancer stem cells to enhance efficacy and reverse the resistance of immunotherapy [9].

Nelfinavir is a first-generation human immunodeficiency virus (HIV) protease inhibitor that was approved by FDA against HIV-1 and HIV-2 infections [11, 12]. Recently, evidence demonstrates the multifaceted anti-cancer properties of nelfinavir including cell proliferation inhibition [13, 14], induction of apoptosis [15, 16] and anti-angiogenesis [17, 18] in multiple cancer types. The promising preclinical anti-cancer efficacy of nelfinavir prompted its clinical trials as monotherapy or in combination with other therapeutics to treat cancer. As monotherapy, nelfinavir benefited patients with liposarcoma [19] and multiple advanced solid tumors [20]. Nelfinavir also demonstrated a positive efficacy in combination with radiochemotherapies to treat solid tumor, such as non-small cell lung cancer (NSCLC) [21], colorectal cancer [22], adenoid cystic head and neck carcinoma [23], pancreatic cancer [24], and advanced hematologic malignancies [25–27]. Mechanically, the cytotoxicity of nelfinavir against cancer cells may derive from the modulation of multiple signaling pathways, including activation of endoplasmic reticulum (ER) stress and unfolded protein response (UPR) [15, 28, 29], induction of autophagy [15, 30], inhibition of proteasomes [28, 31], as well as inducing oxidative stress and targeting mitochondria [16, 32]. Nelfinavir is the most important anti-neoplastic drug of the protease inhibitor family. Repositioning nelfinavir for cancer therapeutics is desirable due to its established knowledge of safety, pharmacokinetics, and adverse events.

Herein, we report that nelfinavir can induce ferroptosis in the HCC cells. Mechanistically, our findings support an indispensable role of ER stress in nelfinavir-induced ferroptosis; and our data further suggest that downstream of ER stress, autophagic degradation of GPX4 and downregulation of SLC7A11 and ferritin, the activation of the Nrf2/HO-1 signaling pathway and resultant increases in Fe^2+^, as well as mitochondrial injury and resultant increases in mitochondrial superoxide may contribute to the induction of ferroptosis by nelfinavir. Moreover, we have found nelfinavir can potentiate the induction of ferroptosis by sorafenib likely through synergistic downregulation of SLC7A11 and GPX4. In vivo HCC xenograft tests further confirm that nelfinavir markedly suppresses HCC tumor growth and this tumor suppressing effect of nelfinavir can be further exacerbated by co-administration of sorafenib. Our current study sheds light on developing potential therapeutic strategies for HCC, particularly advanced and unresectable HCC, through repurposing nelfinavir.

## Results

### Nelfinavir promotes ferroptosis in HCC cell lines

**Fig. 1:**
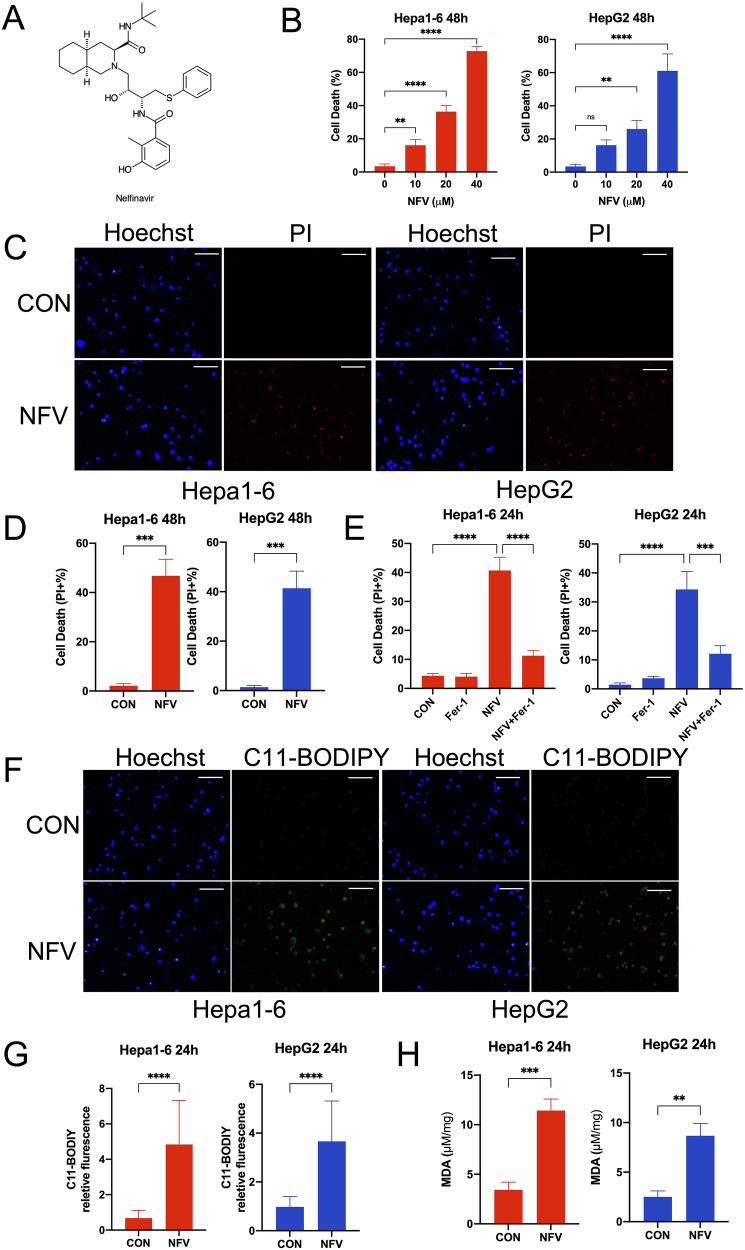
Nelfinavir (NFV) induces ferroptosis. **A** The chemical structure of nelfinavir. **B** Cell death of Hepa1-6 (*n* = 6) and HepG2 (*n* = 6) cells were measured by CCK-8 assay after treatment with the indicated concentration of NFV for 48 h. **C**, **D** Cell death of Hepa1-6 (*n* = 3) and HepG2 (*n* = 3) cells were measured by quantifying the percentage of propidium iodide (PI) positive cells after treatment with NFV (40 µM) for 48 h. **E** The percentage of PI positive cells were compared after Hepa1-6 (*n* = 3) and HepG2 (*n* = 3) cells were treated with ferrostatin-1 (Fer-1, 1 μM), NFV (40 μM), or NFV (40 μM) plus Fer-1 (1 μM) for 24 h. **F**, **G** Nelfinavir-induced lipid peroxidation was quantified using C11-BODIPY fluorescent probes in Hepa1-6 (*n* = 3) and HepG2 (*n* = 3) cells after 24 h treatment. **H** Quantification of cellular MDA levels in Hepa1-6 (*n* = 3) and HepG2 (*n* = 3) cells after 24 h exposure of NFV (40 μM). Scale bar=100 μm. Data were presented as Mean ± SD. Differences were assessed by one-way ANOVA followed by Tukey’s tests. **p < 0.01, ***p < 0.001, and ****p < 0.0001.

To explore whether ferroptosis is a cytotoxic mechanism of nelfinavir in HCC cells, we first tested the cytotoxic effect of nelfinavir on HCC cell lines. Hepa1-6 and HepG2 were exposed to increasing concentrations (0, 10, 20, 40 µM) of nelfinavir for 48 h and the cell death assessment via the CCK-8 assay showed that nelfinavir significantly increased the cell death of both cell lines in a dose dependent manner (Fig. 1B). The PI staining of the 40 μM nelfinavir-treated cells were consistent with the CCK-8 assay by showing that a significant percentage of cells positive for PI (Fig. 1C, D). Importantly, ~60–75% of the cell death were rescued by the ferroptosis inhibitor, Fer-1 (Fig. 1E). Given that ferroptosis is driven by accumulation of lipid peroxidation products, we used C11-BODIPY (a fluorescent lipid peroxidation reporter) fluorescence and MDA (a major secondary oxidation products of peroxidized PUFA) concentration to quantify the level of lipid peroxidation. We found that nelfinavir induced marked lipid peroxidation in Hepa1-6 and HepG2 cells by showing a significant increase of green fluorescence of C11-BODIPY and MDA concentration (Fig. 1F–H). Together, our results indicate that nelfinavir induces ferroptosis in HCC cell lines.

### Nelfinavir downregulates the GPX4-GSH system

**Fig. 2:**
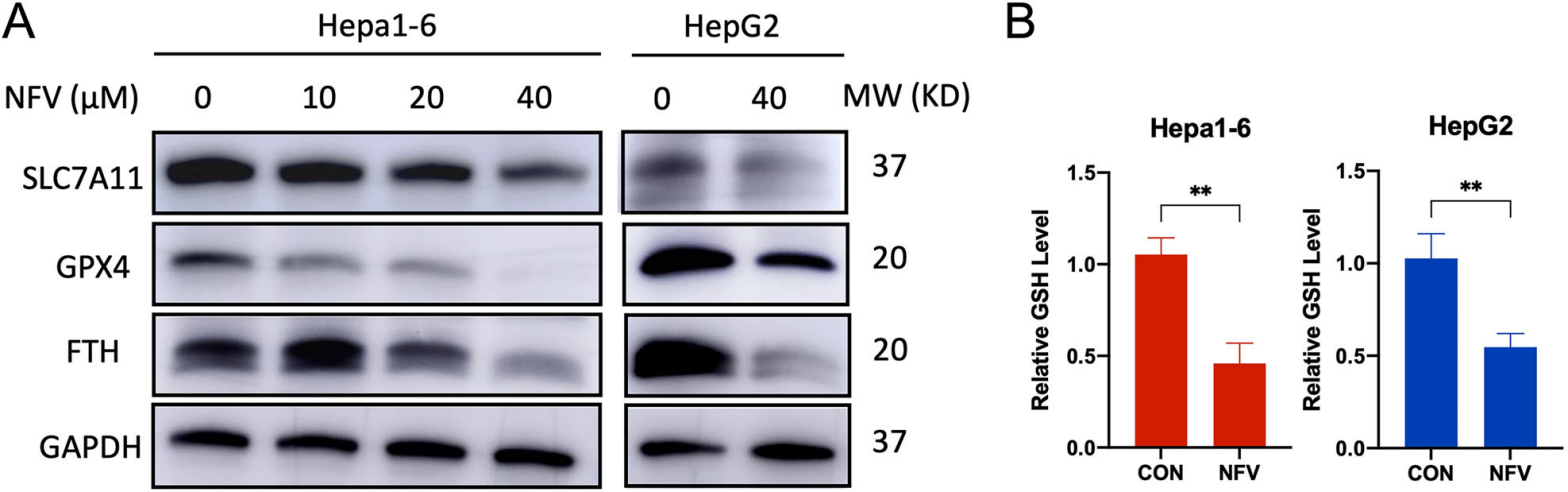
Nelfinavir (NFV) downregulates the System Xc-/GSH/GPX4 pathway. **A** Western blot analyses for SLC7A11, GPX4, FTH (ferritin heavy chain), and GAPDH proteins in Hepa1-6 (*n* = 3) and HepG2 (*n* = 3) cells 24 h after NFV exposure. **B** Quantification of cellular GSH levels in Hepa1-6 (*n* = 3) and HepG2 (*n* = 3) cells after NFV (40 µM) treatment for 24 h. Data were presented as Mean ± SD. Differences were assessed by unpaired *t* test. **p < 0.01.

GPX4, cooperating with its cofactor, reduced glutathione (GSH), acts as a phospholipid hydroperoxidase, directly reducing PLOOHs to their corresponding phospholipid alcohols. The biosynthesis of GSH is highly dependent on System Xc^−^ mediated uptake of cystine, followed by its reduction to cysteine in the cytosol. The System Xc^−^, GSH and GPX4 synergistically orchestrate an adapted antioxidant system against ferroptosis. Targeting System Xc- by agents such as sulfasalazine and erastin [33, 34], or suppressing GPX4 by, for example, RSL3 [35] instigates ferroptosis. So next, we investigated whether nelfinavir would dysregulate the System Xc^−^-GPX4-GSH system. We treated Hepa1-6 cells with escalating concentrations of nelfinavir for 24 h. As shown in Fig. 2A, nelfinavir significantly reduced the protein level of transporter subunits of System Xc^−^, solute carrier family 7 member 11 (SLC7A11), GPX4 and iron storage protein, ferritin (FTH) in a dose dependent manner. Similar decreases were also observed in HepG2 cells after 24 h of exposure to 40 μM nelfinavir (Fig. 2A). A significant depletion of GSH was also observed after the cells were exposed to 40 μM nelfinavir for 24 h (Fig. 2B). Collectively, these results demonstrate that nelfinavir induces downregulation of the GPX4-GSH system.

### Nelfinavir promotes the autophagic degradation of GPX4

**Fig. 3:**
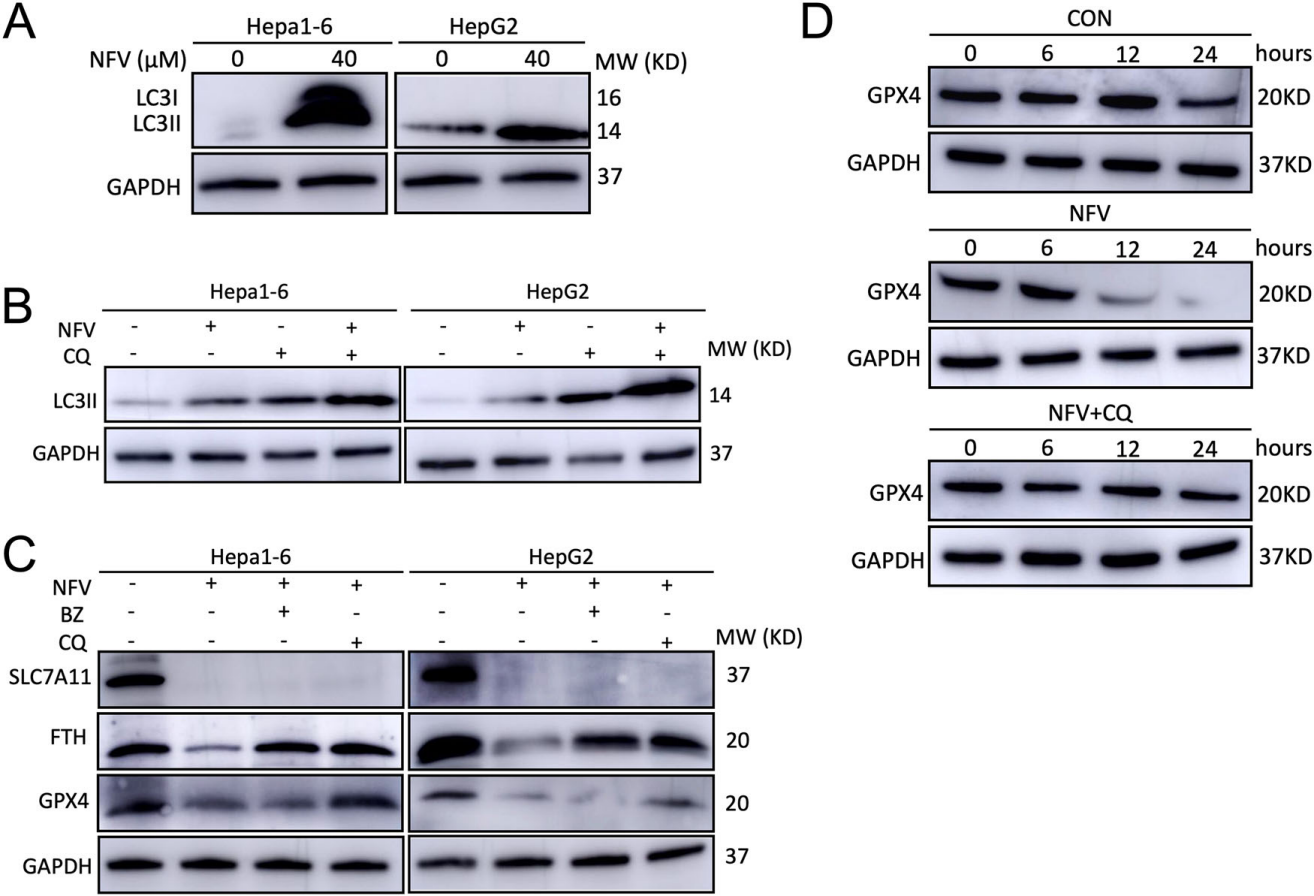
Nelfinavir (NFV) promotes the autophagic degradation of GPX4. **A** Western blot analysis for LC3 in Hepa1-6 (*n* = 3) and HepG2 (*n* = 3) cells after 24 h of NFV treatment. **B** LC3-II flux assay in Hepa1-6 (*n* = 3) and HepG2 (*n* = 3) cells treated with NFV or control. **C** Western blot analyses for SLC7A11, FTH (ferritin heavy chain), GPX4, and GAPDH in Hepa1-6 (*n* = 3) and HepG2 (*n* = 3) cells treated with nelfinavir alone, or in combination of either bortezomib (BZ) or chloroquine (CQ) for 24 h. **D** Cycloheximide chase assays of GPX4 in Hepa1-6 (*n* = 3) cells treated with DMSO (CON), NFV (40 µM), or NFV (40 µM) combined with CQ (40 µM). Cells were harvested for total protein extraction immediately before (0) or 6, 12, and 24 h after the initiation of cycloheximide treatment. Data were presented as Mean ± SD. Differences were assessed by one-way ANOVA followed by Tukey’s tests. ***p < 0.001 and ****p < 0.0001.

Next, we sought to explore the mechanism by which nelfinavir downregulates System Xc−/GPX4. The degradation of GPX4 proteins is regulated by the ubiquitin-proteasome system (UPS) [36] and autophagy [37]. We then investigated whether nelfinavir promoted the protein degradation of GPX4. Firstly, we tested whether nelfinavir would enhance macroautophagy in Hepa1-6 and HepG2 cells (Fig. 3A). After 24 h treatment, nelfinavir significantly increased the protein level of LC3II, a commonly used marker of autophagic vacuoles. The subsequent autophagic flux experiment confirmed that nelfinavir induced autophagy in the two HCC cell lines (Fig. 3B), suggesting increased autophagic degradation may be responsible for the downregulation of GPX4 by nelfinavir. Indeed, our further experiments revealed that inhibition of lysosomes by chloroquine (CQ), but not inhibition of proteasome by bortezomib (BZ), dramatically rescued the protein abundance of GPX4 in Hepa1-6 and HepG2 cells treated with nelfinavir (Fig. 3C). Meanwhile, the inhibition of either proteasome or lysosome partially restored the protein level of Ferritin. However, neither proteasome inhibition nor lysosome blockade restored the protein level of SLC7A11, which suggested nelfinavir may downregulate SLC7A11 at the translational or transcriptional level. To further confirm that nelfinavir promoted the autophagic degradation of GPX4, we performed cycloheximide chase experiments to examine the protein decay of GPX4 after cycloheximide-exerted inhibition of protein synthesis in Hepa1-6 cells (Fig. 3D). Compared to control, nelfinavir dramatically increased the degradation rate of GPX4, which was decreased by adding CQ to inhibit lysosomes. Collectively, our results suggest that nelfinavir promotes autophagic degradation of GPX4.

### ER stress contributes to nelfinavir-induced ferroptosis in HCC cells

**Fig. 4:**
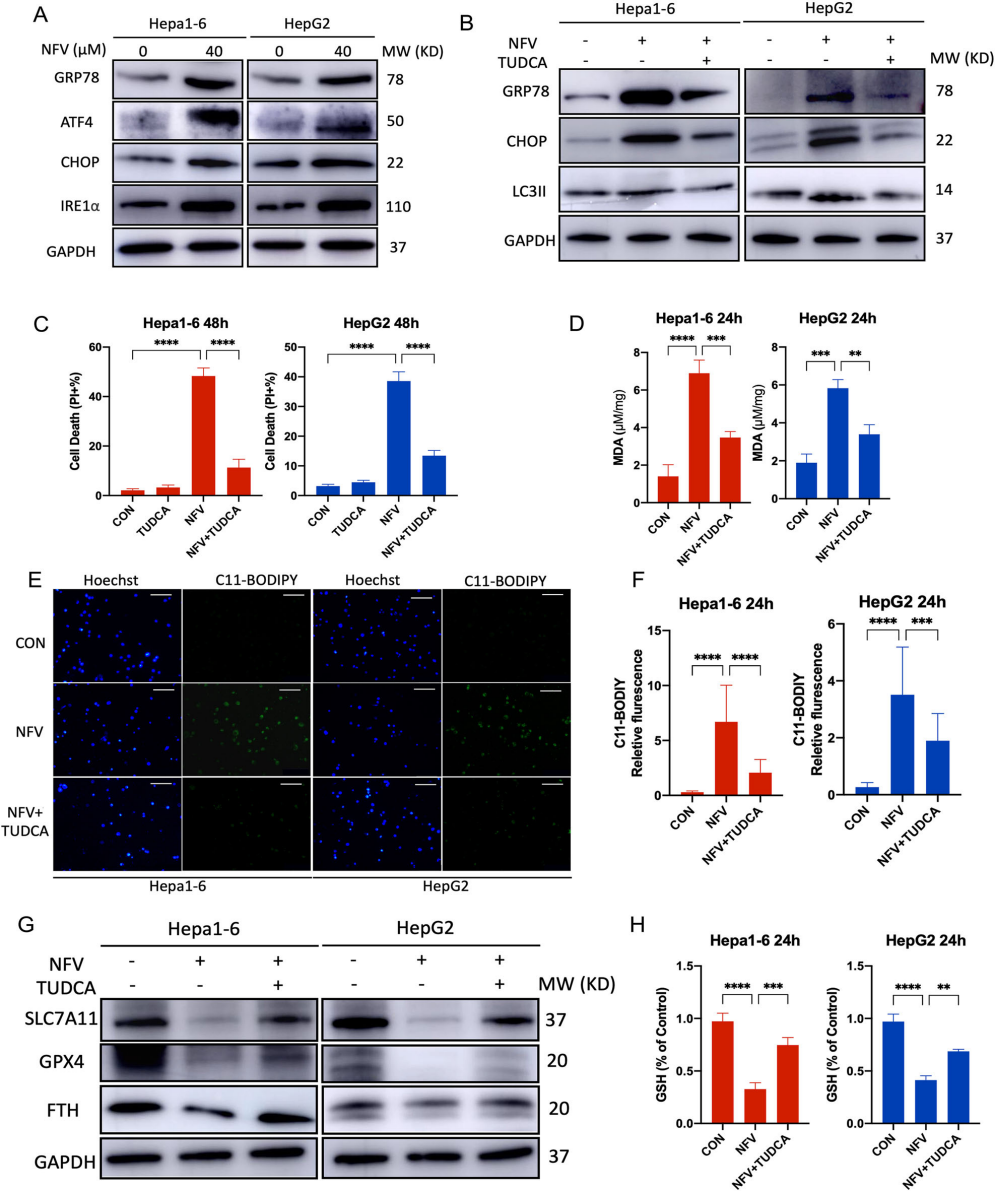
Nelfinavir (NFV) activates ER stress. **A** Western blot analyses for ER stress related protein GRP78, ATF4, CHOP, and IRE1α in Hepa1-6 (*n* = 3) and HepG2 (*n* = 3) cells after 24 h treatment of NFV (40 µM). **B** Effect of TUDCA (200 μM) on NFV-induced upregulation of ER stress associated protein of GRP78 and CHOP, and autophagy associated protein LC3 after 24 h treatment. *n* = 3 independent replicates for Hepa1-6 and HepG2 cells respectively. **C** Effect of TUDCA on NFV-induced cell death after 48 h treatment. *n* = 3 independent replicates for Hepa1-6 and HepG2 cells respectively. **D** Effect of TUDCA on NFV-induced lipid peroxidation, quantified by MDA level after 24 h treatment. *n* = 3 independent replicates for Hepa1-6 and HepG2 cells respectively. **E**, **F** Effect of TUDCA on NFV-induced lipid peroxidation, quantified by C11-BODIPY fluorescence probe after 24 h treatment. *n* = 3 independent replicates for Hepa1-6 and HepG2 cells respectively. Scale bar=100 μm. **G** Effect of TUDCA on NFV-mediated downregulation of SLC7A11, GPX4, Ferritin protein expression after 24 h treatment. *n* = 3 independent replicates for Hepa1-6 and HepG2 cells respectively. **H** Effect of TUDCA on NFV-mediated decrease of GSH level after 24 h treatment. *n* = 3 independent replicates for Hepa1-6 and HepG2 cells respectively. Data were presented as Mean ± SD. Differences were assessed by one-way ANOVA followed by Tukey’s tests. **p < 0.01, ***p < 0.001, and ****p < 0.0001.

ER stress induces distinct cell death by activating the three key transmembrane sensors of UPR, protein kinase R-like endoplasmic reticulum kinase (PERK), Inositol-requiring enzyme type 1α (IRE1α), and activating transcription factor 6 (ATF6). Recent studies have unraveled the important roles of ER stress in ferroptosis; ER stress not only can result from the process of ferroptosis but also contributes to ferroptosis [38, 39]. Meanwhile, crosstalk between ER stress and autophagy has been well illustrated. ER stress has been reported to stimulate autophagy by integrating all UPR branches [40]. Considering the potent ER stress-modulating effects of nelfinavir [12], we tested whether nelfinavir facilitated ferroptosis through induction of ER stress in HCC cells. Firstly, we found that nelfinavir induced ER stress and activated PERK and IRE1α branches in Hepa1-6 and HepG2 cells after 24 h treatment (Fig. 4A). To further investigate whether ER stress was involved in nelfinavir-induced ferroptosis, we subjected Hepa1-6 and HepG2 cells to co-treatment of TUDCA, a well-known ER stress inhibitor, with nelfinavir. After 24 h exposure, TUDCA significantly alleviated nelfinavir induced ER stress by showing reduced protein expression of GRP78 and CHOP, while a remarkable decrease of LC3II was also observed (Fig. 4B). TUDCA dramatically attenuated nelfinavir-induced cell death (Fig. 4C) and lipid peroxidation (Fig. 4D–F). Meanwhile, TUDCA reversed the protein expression of SLC7A11, GPX4 and ferritin that was decreased by nelfinavir in Hepa1-6 and HepG2 cells (Fig. 4G). The GSH level was also restored by TUDCA (Fig. 4H). Collectively, our results demonstrate that ER stress plays a major role in nelfinavir-induced ferroptosis in HCC cells.

### Nelfinavir activates Nrf2/HO-1 pathway via ER stress

**Fig. 5:**
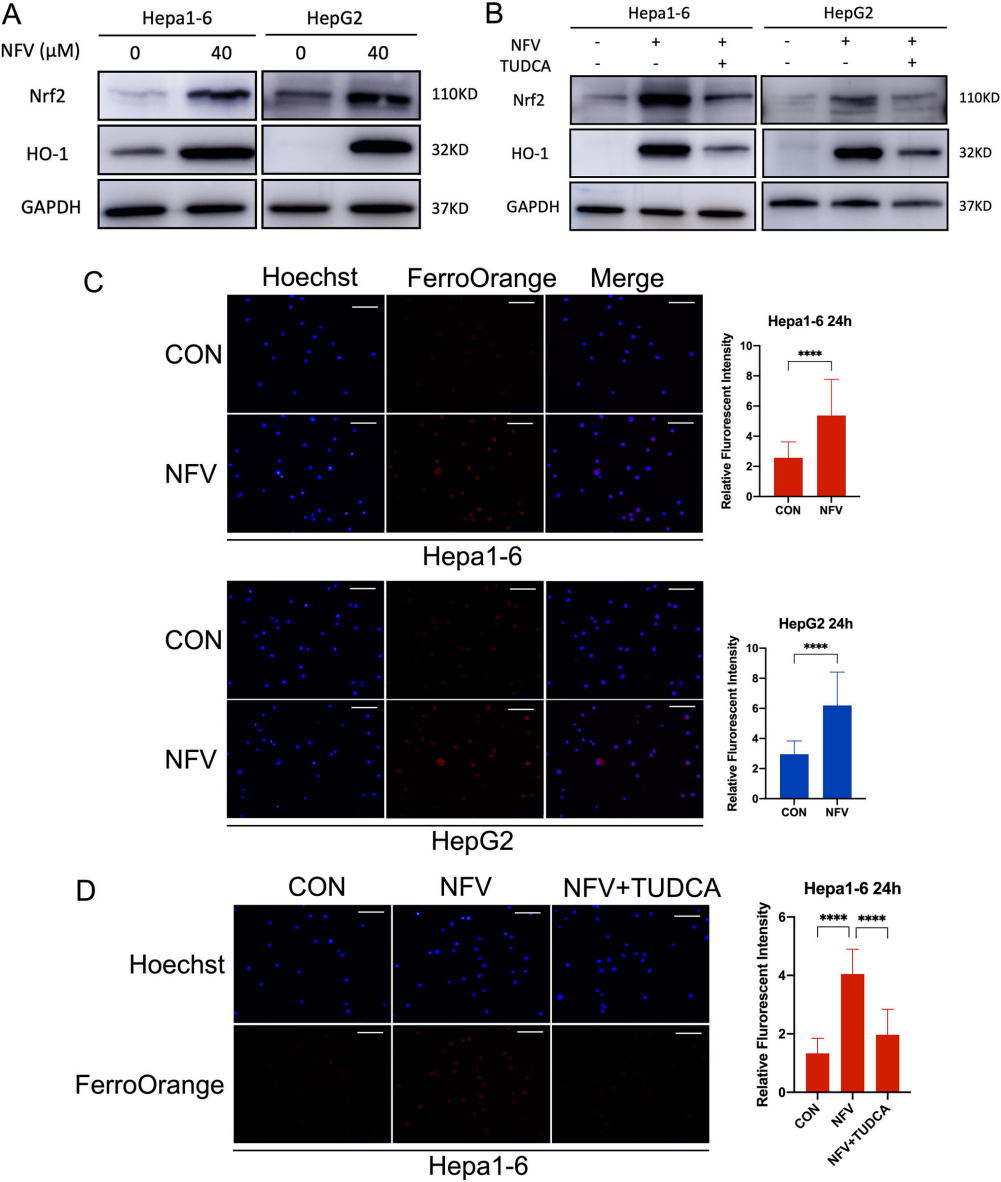
Nelfinavir (NFV) induces activation of Nrf2/HO-1 via ER stress. **A** Western blot analyses for Nrf2 and HO-1 in Hepa1-6 and HepG2 cells after 24 h treatment of NFV (40 µM). **B** Effect of TUDCA (200 μM) on NFV-induced Nrf2 and HO-1 protein expression after 24 h treatment. **C** Measurement of intracellular ferrous ion levels by quantifying FerroOrange fluorescence intensity after 24 h NFV treatment. *n* = 3 independent replicates for Hepa1-6 and HepG2 cells respectively. **D** Effect of TUDCA (200 μM) on NFV-induced ferrous ion levels as reflected by changes in FerroOrange fluorescence intensity after 24 h treatment in Hepa1-6 cells (*n* = 3). Scale bar=100 μm. Data were presented as Mean ± SD. Differences were assessed by unpaired *t* tests. **p < 0.01 and ****p < 0.0001.

Nuclear factor erythroid 2-related factor 2 (Nrf2) is a master transcriptional factor that translocates into the nucleus to transactivate its target genes in response to oxidative stress and ER stress [39]. As one of the target genes, heme oxygenase 1 (HO-1) is an ER-anchored enzyme that catalyzes heme into pro-oxidant ferrous iron, carbon monoxide, and antioxidant biliverdin [39]. Accumulated studies have shown that enhanced activation of Nrf2/HO-1signaling promotes ferroptosis [39, 41, 42]. Our results showed that nelfinavir significantly upregulated Nrf2 and HO-1 in Hepa1-6 and HepG2 cells after 24 h exposure (Fig. 5A). Attenuation of ER stress by TUDCA antagonized nelfinavir-related increase of Nrf2 and HO-1 (Fig. 5B). Our previous results showed that nelfinavir promoted the degradation of ferritin. The combined effect of ferritin downregulation and HO-1 upregulation may increase the intracellular ferrous level, which facilitates ferroptosis through the Fenton reaction. To explore this hypothesis, we measured the ferrous level with FerroOrange after 24 h treatment of nelfinavir (Fig. 5C). Compared to control, nelfinavir increased the fluorescent intensity of FerroOrange in Hepa1-6 and HepG2 cells. In addition, suppressing ER stress by TUDCA significantly decreased the ability of nelfinavir to increase the ferrous level as reflected by the changes in FerroOrange fluorescent intensity (Fig. 5D). Collectively, these results are consistent with the contention that activation of Nrf2/ HO-1 pathway by ER stress is involved in nelfinavir-induced ferroptosis in HCC cells.

### Nelfinavir impairs mitochondrial membrane potential and promotes superoxide production in HCC cells

**Fig. 6:**
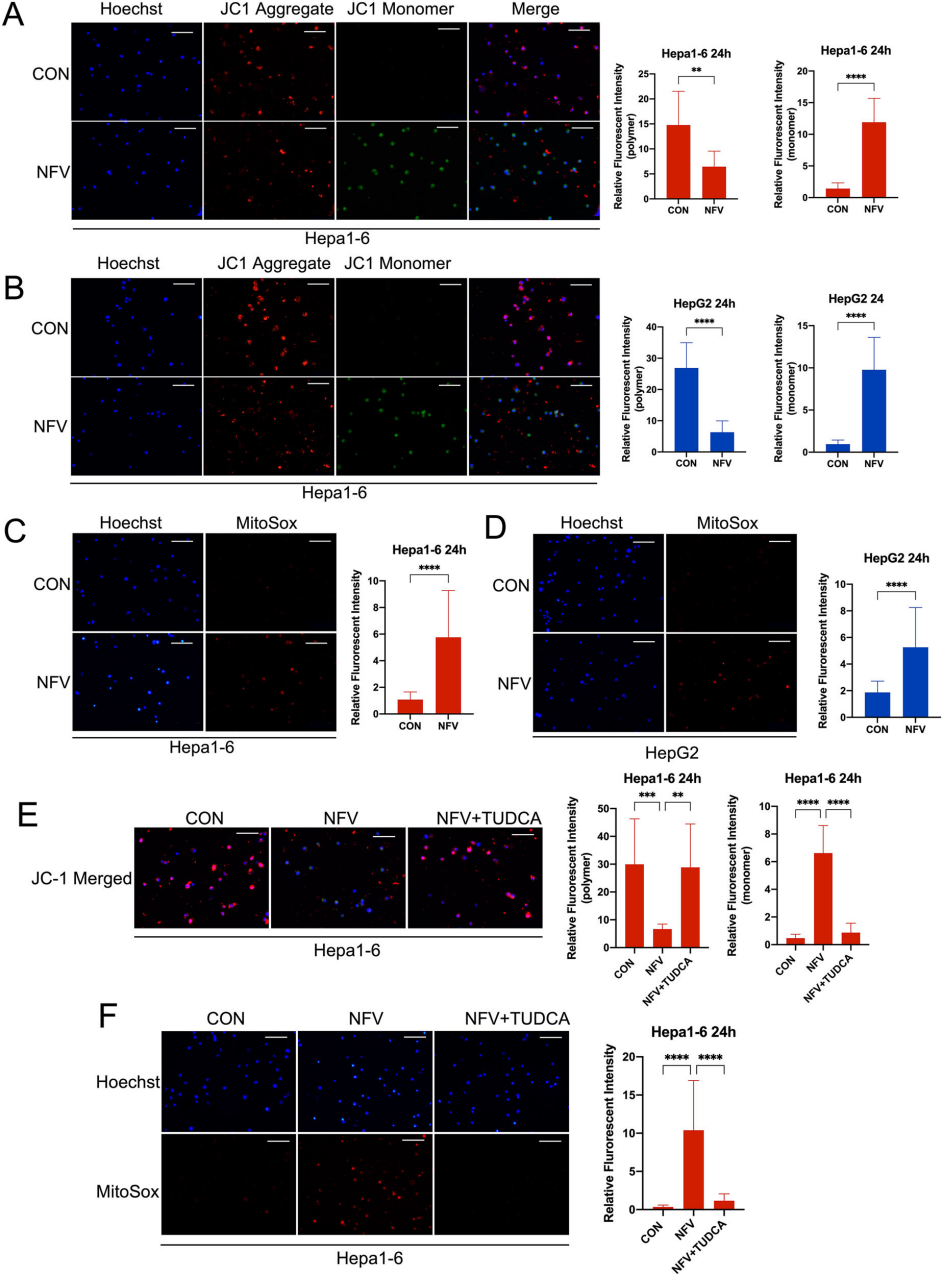
Nelfinavir (NFV) impairs mitochondrial membrane potential and promotes superoxide production. Hepa1-6 cells (*n* = 3) (**A**) and HepG2 cells (*n* = 3) (**B**) were treated with NFV (40 µM) for 24 h and incubated with JC-1 probe for 30 min before imaged. The fluorescence of JC-1 polymers and monomers were quantified, and the relative intensity is shown in the associated bar graphs. Hepa1-6 cells (*n* = 3) (**C**) and HepG2 cells (*n* = 3) (**D**) were treated with NFV (40 µM) for 24 h and incubated with MitoSox probe for 30 min before imaged. The fluorescence of MitoSox was quantified, and the relative intensity is shown in the associated bar graphs. The effects of TUDCA on the fluorescence of JC-1 polymers and monomers (**E**) and the fluorescence of MitoSox (**F**) in Hepa1-6 cells that were treated with NFV for 24 h. Scale bar=100 μm. Data were presented as Mean ± SD. Differences were assessed by unpaired *t* test. **p < 0.01 and ****p < 0.0001.

Recent studies unraveled the role of mitochondria in ferroptosis. The active involvement of mitochondria in bioenergetic, biosynthetic and ROS regulation contributes to its pro-ferroptosis function [43]. To investigate the effect of nelfinavir on mitochondrial homeostasis, we used JC-1 to measure mitochondrial membrane potential, loss of which indicates mitochondrial dysfunction. In healthy cells, JC-1 enters and accumulates in the negatively charged mitochondria and spontaneously form red fluorescent aggregates. By contrast, JC-1 will retain its green fluorescence if mitochondria are hypo-polarized due to increased membrane permeability [44]. Our results showed that nelfinavir exposure significantly decreased the red fluorescence of JC-1 aggregates but increased the green fluorescence of JC-1 monomer in Hepa1-6 and HepG2 cells (Fig. 6A, B). This result suggests that nelfinavir impairs the mitochondrial membrane potential, leading to mitochondrial dysfunction. As the major source of intracellular reactive oxygen species (ROS), mitochondria-associated superoxide likely contributes to ferroptosis through promoting lipid peroxidation [43]. We next tested the effect of nelfinavir on the production of mitochondrial superoxide with the MitoSox probe. We found that nelfinavir significantly increased the red fluorescence of MitoSox in Hepa1-6 and HepG2 cells (Fig. 6C, D). Moreover, the ER stress inhibitor, TUDCA, significantly restored the mitochondrial membrane potential and attenuated the production of mitochondrial superoxide that were induced by nelfinavir in Hepa1-6 cells (Fig. 6E, F). Collectively, these results suggest that nelfinavir impairs mitochondrial function and promotes production of superoxide in an ER stress-dependent manner.

### Nelfinavir potentiates sorafenib-induced ferroptosis in HCC cells

**Fig. 7:**
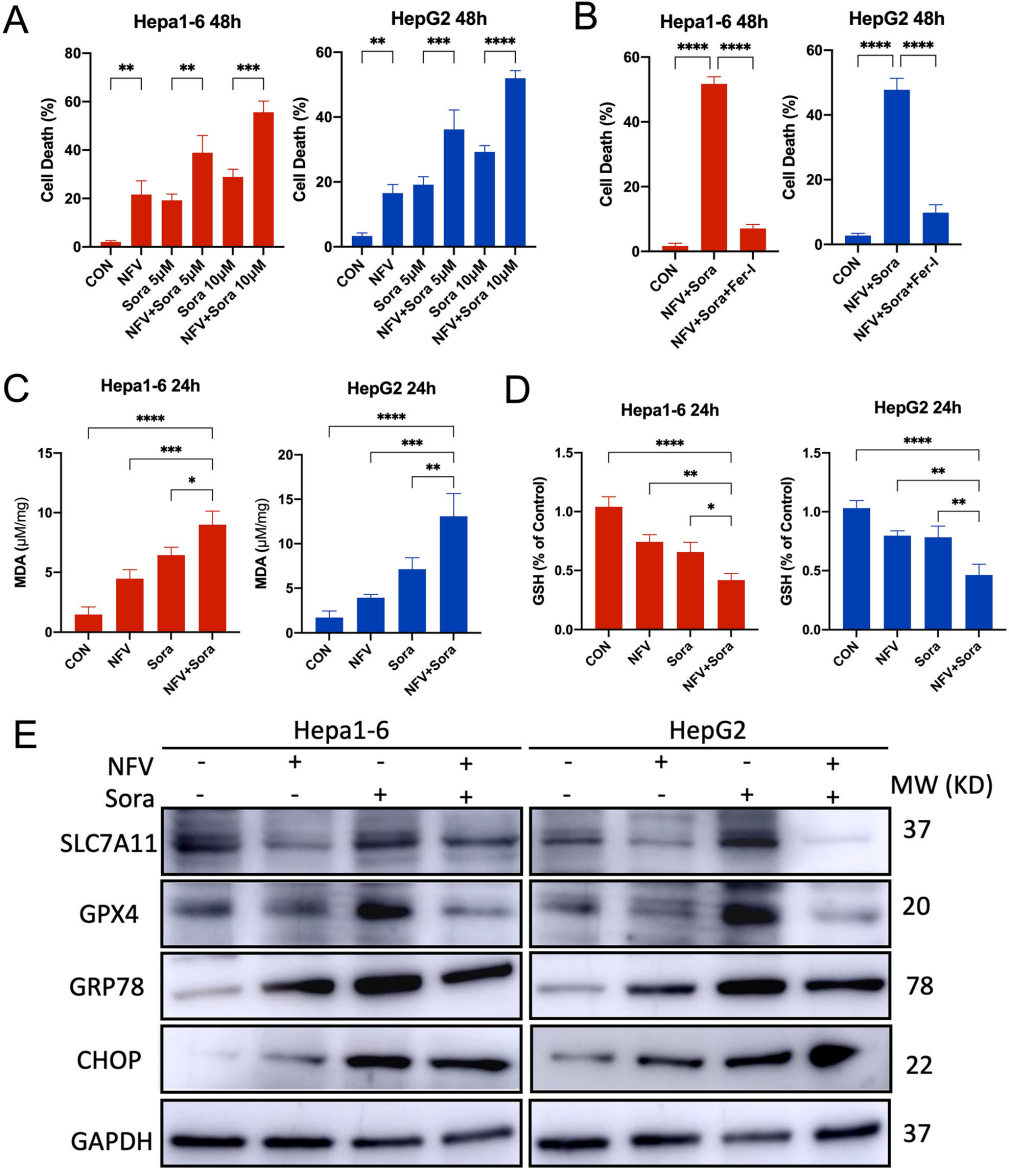
Nelfinavir (NFV) potentiates sorafenib-induced ferroptosis. **A** Cell death was measured using the CCK-8 assay in Hepa1-6 (*n* = 6) and HepG2 (*n* = 6) cells treated with sorafenib (Sora, 5 μM and 10 μM) with or without NFV (20 μM) for 48 h. **B** Cell death was measured using the CCK-8 assay in Hepa1-6 (*n* = 6) and HepG2 (*n* = 6) cells treated with combined NFV (20 μM) and sorafenib (Sora, 10 μM) with or without Fer-1 (1 μM) for 48 h. The contents of the cellular MDA (**C**) and GSH (**D**) in Hepa1-6 and HepG2 cells treated with sorafenib (10 μM) and/or NFV (20 μM) for 24 h were determined. **E** Western blot analyses for SLC7a11, GPX4, GRP78, and CHOP after 24 h exposure of sorafenib (10 μM) and/or NFV (20 μM). *n* = 3 independent replicates for Hepa1-6 and HepG2 cells respectively. Data were presented as Mean ± SD. Differences were assessed by one-way ANOVA followed by Tukey’s tests. *p < 0.05, **p < 0.01, ***p < 0.001, and ****p < 0.0001.

Sorafenib, a tyrosine kinase inhibitor, was approved for advanced HCC in 2007. It was demonstrated as a strong ferroptosis inducer by inhibiting System Xc- [45]. Thus, we explored whether there is possibility for nelfinavir to enhance the tumoricidal efficacy of sorafenib. Comparing to sorafenib (5 µM and 10 µM) alone, addition of nelfinavir (20 µM) significantly increased the induction of cell death in Hepa1-6 and HepG2 cells by sorafenib (Fig. 7A). To analyze whether the combined effects are synergistic, we used the Calcusyn program to calculate the combination index (CI) values of NFV/Sora cotreatments. For a specific drug association, a CI < 1 indicates synergism, CI = 1 indicates additivism, whereas CI > 1 indicates antagonism [46]. The CI values was 0.698 and 0.432 for Hepa1-6 cells when nelfinavir (20 µM) was in combination with 5 µM and 10 µM sorafenib, respectively. For HepG2 cells, the CI values were 0.887 and 0.495 when nelfinavir (20 µM) was combined with 5 µM and 10 µM sorafenib, respectively. The CI values indicate a synergism between nelfinavir and sorafenib in the applied doses to treat the HCC cells.

The combined treatment induced cell death was nearly completely rescued by Ferrostatin-1 (Fer-1), a potent and selective inhibitor of ferroptosis (Fig. 7B). Meanwhile, the increases in the level of MDA, an indicator of lipid peroxidation (Fig. 7C), and decreases in GSH levels (Fig. 7D) were markedly greater in cells treated with both nelfinavir and sorafenib than those treated with each alone. Our western blot analyses showed that combined treatment of nelfinavir and sorafenib further decreased the protein expression of SLC7A11 and GPX4. However, the combination did not further increase the expression of GRP78 and CHOP (Fig. 7E). Additionally, similar results were also found when nelfinavir was combined with RSL3 (a ferroptosis inducer that inhibits GPX4 directly) in Hepa1-6 and HepG2 cells (Fig. S1). Collectively, our results indicate that nelfinavir enhances sorafenib induced ferroptosis likely by further dampening the GPX4-GSH system.

### Nelfinavir and nelfinavir plus sorafenib inhibit HCC tumor growth in vivo

**Fig. 8:**
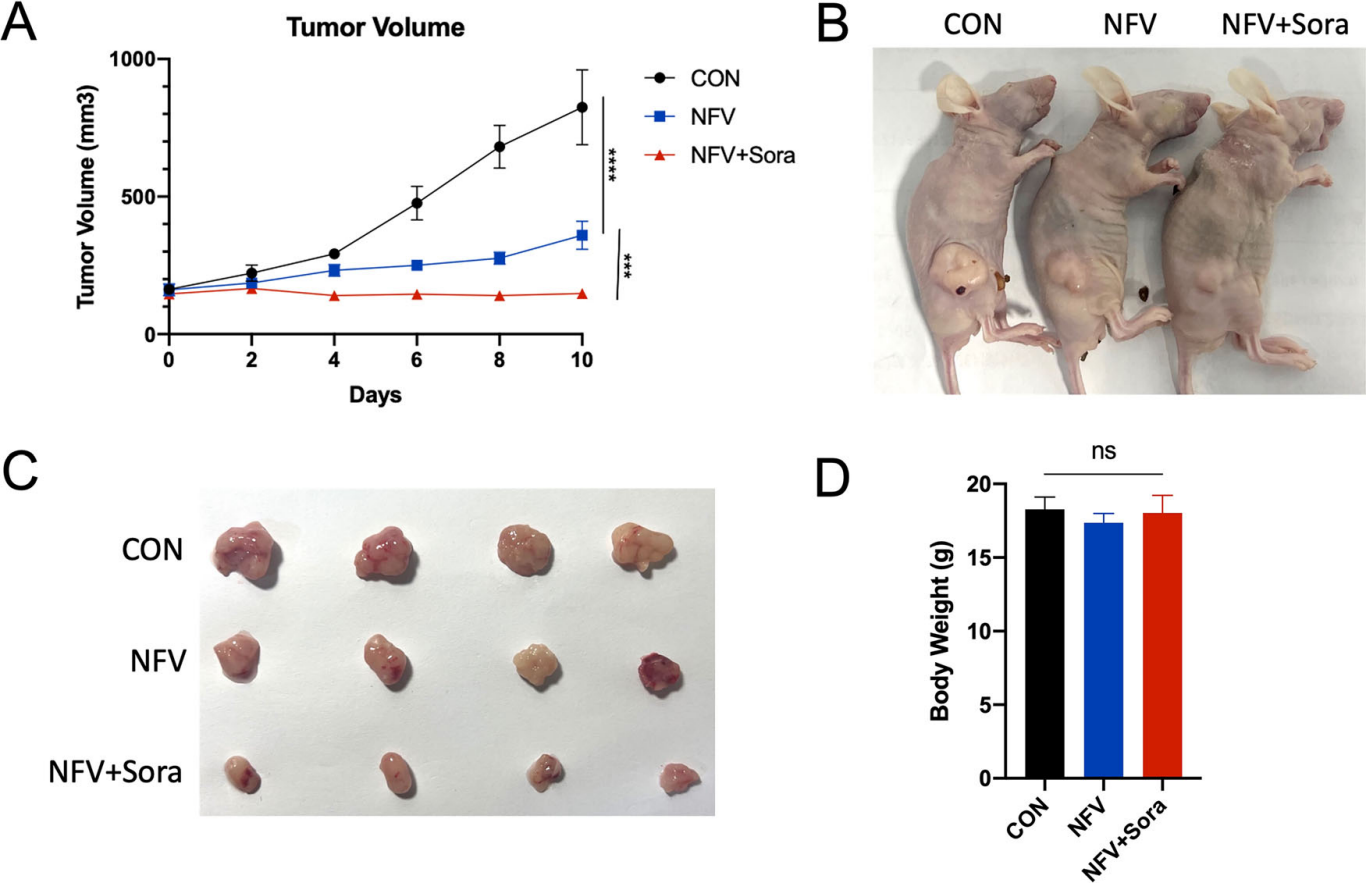
Effects of nelfinavir (NFV) and nelfinavir plus sorafenib (Sora) on tumor growth of HCC xenografted mice. BALB/c nude mice bearing the Hepa1-6 subcutaneous xenografts (*n* = 4 mice per group) received vehicle, NFV (i.p., 50 mg/kg), and NFV+Sora (i.p., 50 mg/kg+30 mg/kg) as indicated, once daily. **A** Tumor volume progression, **B** representative tumor bearing mouse images, **C** representative tumor images, and **D** body weights are shown. Data were presented as Mean ± SD. Differences were assessed by one-way ANOVA followed by Tukey’s tests. ***p < 0.001, and ****p < 0.0001.

To evaluate the effects of nelfinavir and nelfinavir plus sorafenib on HCC tumor growth, we established a Hepa1-6 subcutaneous xenograft model in BALB/c nude mice and injected them with vehicle (PBS), nelfinavir (50 mg/kg), or nelfinavir plus sorafenib (50 mg/kg+30 mg/kg) once per day. Tumor volume and body weight were monitored every other day. Results showed that nelfinavir significantly suppressed tumor growth (Fig. 8A–C). The combined treatment of nelfinavir and sorafenib further retarded tumor growth (Fig. 8A–C). Neither nelfinavir nor nelfinavir plus sorafenib showed discernible effects on the body weight of the mice (Fig. 8D). Our results demonstrate that nelfinavir and nelfinavir plus sorafenib strongly suppress tumor growth in HCC xenograft model.

## Discussion

**Fig. 9:**
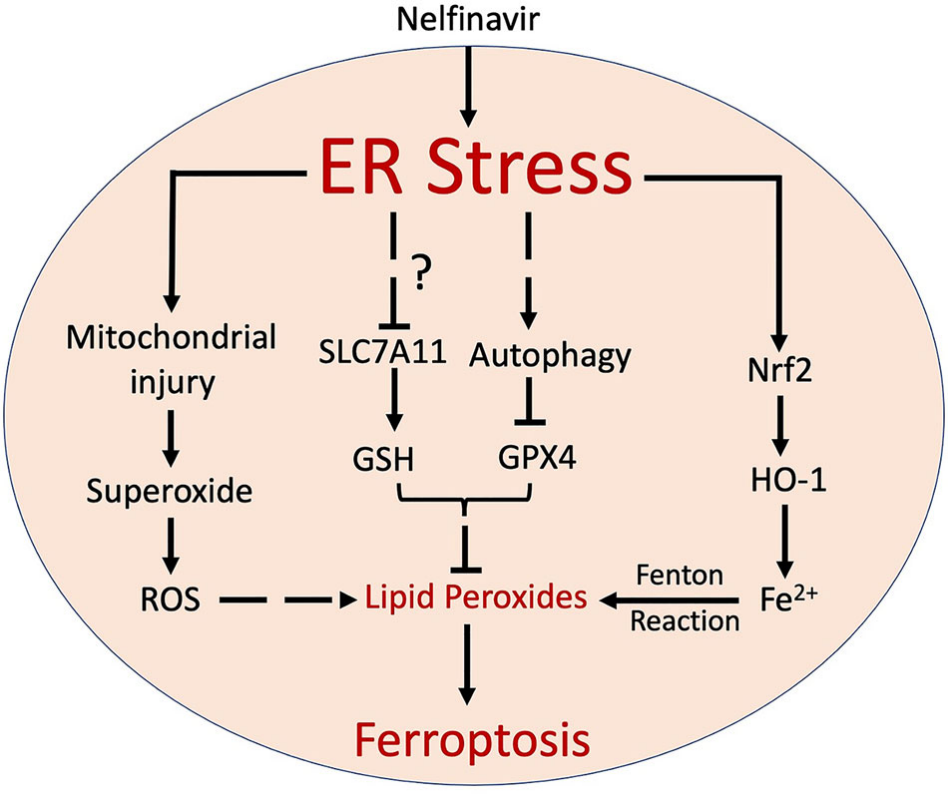
A model for nelfinavir to induce ferroptosis in HCC cells. Nelfinavir activates ER stress, leading to downregulation of GPX4/GSH system, upregulation of Nrf2/HO-1 axis, and mitochondrial injury, which results in ferroptosis in HCC cells. Dashed line, multiple signaling steps. Question mark, unknown mechanism.

Development of novel therapeutic strategies that improve HCC prognosis is still urgently demanded. Ferroptosis, a recently characterized form of programmed cell death that is driven by iron dependent lipid peroxidation, has been highlighted as an efficient anti-tumoral mechanism in eradication of cancer cells, particularly of malignant cancer cells that are resistant to conventional therapies [8, 47]. Ferroptosis induction suppresses the growth and progression of hepatocellular carcinoma and promotes chemotherapeutic sensitization [48]. Hence, small molecules that promote ferroptosis should benefit HCC therapy. Here, we have found nelfinavir as a novel ferroptosis inducer that increases the level of cellular lipid peroxidation, MDA, and the labile iron pool, but downregulates the GSH level in Hepa1-6 and HepG2 cells. Mechanically, nelfinavir promotes ER stress-dependent downregulation of three important ferroptosis regulators SLC7A11, GPX4 and ferritin. Meanwhile, nelfinavir-induced ER stress also upregulated the Nrf2/HO-1 axis in Hepa1-6 and HepG2 cells. The resultant deregulation of antioxidant defense and upregulation of ferrous ions promote ferroptosis in HCC cell lines (Fig. 9).

Nelfinavir is a potent ER stress modulator. Our experimental results indicate that nelfinavir induced ER stress, triggers the deregulation of the GPX4-GSH system in HCC cells. Occurrence of ER stress and induction of ferroptosis can be intertwined [38, 49]. Ferroptosis inducers like sorafenib, dihydroartemisinin (DHA), erastin and RSL3 have been shown to induce ER stress [50]. PERK signaling activation was reported to upregulate p53 which downregulated transcription of SLC7A11 [51, 52]. ATF3, a downstream factor of PERK/ eIF2α branch, was also reported to repress SLC7A11 in a p53-independent manner [53, 54]. Decreased SLC7A11 resulted in inhibition of system Xc- activity, depletion of GSH, and ferroptosis. In addition, activation of PERK-eIF2α-ATF4-CHOP signaling pathway was shown to participate in ferroptosis induction and, conversely, inhibition of the PERK-eIF2α-ATF4-CHOP pathway attenuated ferroptosis [55–57]. CHAC1, a downstream factor of the ATF4-CHOP pathway, decreased GSH level and sensitized cells to lipid peroxidation [38, 58]. On the other hand, ER stress responses also impair GPX4 activity. IRE1α upregulated Xbp1 to activate Gα_12_, whose overexpression downregulated GPX4 [59]. ER stress also accelerated degradation of GPX4 via chaperone mediated autophagy (CMA) [60]. In our study, we found that the protein level of SLC7A11 was significantly decreased by nelfinavir in a ER stress-dependent manner. Neither inhibition of proteasomes nor suppressing lysosomes restored the protein level of SLC7A11, which suggests that nelfinavir possibly decreases the synthesis of SLC7A11 at transcriptional or translational levels. Whether nelfinavir induces ferroptosis through p53 dependent or independent signaling pathway needs further evaluation. Our results also demonstrate that nelfinavir promotes degradation of GPX4 via autophagy. It was reported that enhanced autophagy and ER stress were linked as an upstream activating factor for nelfinavir-associated autophagy [15, 61]. Consistent with previous studies, our results showed that nelfinavir-induced autophagy was alleviated by ER stress inhibitor, TUDCA, the decrease of which upregulated GPX4 expression. Therefore, it is suggested that nelfinavir promotes GPX4 degradation by ER stress-dependent autophagy. However, further experiments are needed to unveil whether a particular chaperone (such as HSC70 or HSP90 [62]) is involved in nelfinavir-induced autophagic degradation of GPX4.

Nrf2 is a stress-inducible transcriptional factor that plays multifaceted roles in antioxidative responses. Activated Nrf2 translocates to the nucleus and directly binds to antioxidant response elements (AREs) to transactivate downstream antioxidant genes, including HO-1, GCLM, SLC7A11, GCLC, FTH1, ATF6, NQO1, GAPDH, ATF3, and Nrf2 itself [39]. A number of Nrf2 targeted genes prevent lipid peroxidation and ferroptosis, including SLC7A11 and GPX4 [63]. However, recent studies have discovered the Nrf2-HO-1 signaling pathway promotes ferroptosis upon ER stress [39]. Activated PERK phosphorylated Nrf2 and activated its transcriptional activity to upregulate HO-1 [39]. HO-1 plays dual role in ferroptosis induction, either cytoprotective effects or detrimental effects. High expression of HO-1 promoted ferroptosis by increasing iron accumulation and ROS production [64–66]. Consistent with previous studies, nelfinavir also functioned as a ferroptosis inducer through activation of the Nrf2-HO-1 signaling pathway. Meanwhile, nelfinavir promoted the degradation of ferritin via the UPS and autophagy. Thus, the combined effect may synergistically increase cellular iron accumulation, facilitating ferroptosis.

Mitochondria participate in governing ferroptosis [43]. Mitochondria-associated ROS contributes to ferroptosis induction through promoting lipid peroxidation. Hydrogen peroxide (H_2_O_2_), converted from mitochondrial superoxide, reacts with ferrous ions to generate hydroxyl radicals, which facilitate the production of PUFA hydroperoxides [43]. Nelfinavir was reported that enhanced oxidative stress and induced mitochondrial damage in cancer cells [16, 67, 68]. Our results indicated that nelfinavir induced mitochondrial dysfunction and superoxide production by showing a loss of mitochondrial membrane potential and increased MitoSox fluorescence. The resultant enhancement of oxidative stress may propagate lipid peroxidation and activate ER stress [69]. Meanwhile, during ER stress, released Ca^2+^ from ER also promotes mitochondrial ROS production [69]. This positive feedback cycle between ER and mitochondria could exacerbate the ferroptosis induced by nelfinavir. In addition, mitochondrial DNA leakage and oxidative stress could activate the STING pathway and triggered ferroptosis [70–72]. Moreover, STING activation also induces ER stress [73, 74]. These previous findings suggest that STING might be a regulator in the crosstalk between ER stress and ferroptosis. Our results also demonstrated that nelfinavir dramatically activated STING in HCC cells, and this activation was partially dependent on ER stress (Figs. S2A and 2B). Chemical blockade of STING via C-176 partially restored nelfinavir-triggered decreases in GPX4 (Fig. S2D), which is agreement with a recent report that STING activation induced autophagic degradation of GPX4 [75]. However, our experiment showed inhibition of STING via C-176 did not rescue the cell death (Fig. S2C). Thus, STING’s contribution to nelfinavir’s induction of ferroptosis remains to be further clarified.

Sorafenib, a multi-kinase inhibitor, is the standard of care for advanced HCC [76]. Combined sorafenib with existing clinical drugs, such as everolimus (inhibitor of mTOR), ibrutinib (tyrosine kinase inhibitor), bortezomib (proteasome inhibitor) and ramucirumab (VEGFR2 inhibitor), have been widely tested in preclinical experiments and clinical trials to improve the efficacy of HCC treatment [77–80]. Recent studies identified sorafenib as a ferroptosis inducer [81, 82]. It restricts cystine uptake by inhibiting system Xc-, which prompts ER stress, GSH depletion and iron-dependent lipid peroxidation, and ultimately induces ferroptosis [83]. Our results demonstrated that the combined treatment of nelfinavir together with sorafenib synergistically downregulated protein expression of SLC7A11 and GPX4 in HCC cells and suppressed tumor growth in a subcutaneous xenografted HCC mouse model. Upregulation of SLC7A11 prevents lipid peroxidation, which contributes to acquired ferroptosis resistance of sorafenib [82]. Nelfinavir exacerbated the downregulation of SLC7A11 caused by sorafenib, which makes nelfinavir an ideal drug with a known mechanism of action to combine with sorafenib for HCC treatment. However, multiple downstream factors of UPR, such as ATF4 and Nrf2, promoted the upregulation of SLC7A11 [82]. Considering the strong ER stress modulating effect of nelfinavir, it is still unclear regarding the regulatory mechanism of nelfinavir on SLC7A11.

## Materials and methods

### Cell culture and Reagents

Hepa1-6 and HepG2 cell lines were purchased from SunnCell (Wuhan, China). Both cell lines were cultured in DMEM (Gibco, USA) supplemented with 10% fetal bovine serum (Corning, USA), 1% antibiotics (penicillin 10,000 μ/ml, streptomycin 100 mg/ml) (Hyclone, USA). The cells were maintained at 37 °C in a humidified atmosphere with 5% CO_2_ and were used during their logarithmic growth phase. The drugs used to treat the cell were initially dissolved in DMSO. The manufactures of the drugs are listed as follows: nelfinavir (HY-15287, MCE), Chloroquine (HY-17589A, MCE), Cycloheximide (HY-12320, MCE), Sorafenib (HY-10201, MCE), RSL3 (HY-100218A, MCE) and Tauro ursodeoxycholic acid (TUDCA) (HY-19696A, MCE).

### Assessment of cell viability with CCK-8 assay

The cell death was measured by CCK-8 assay (HY-K0301, MCE). Cells were seeded in 96-well plates in 100 μL medium. After the respective treatment, 10 μL CCK-8 solution was added to each well and incubated at 37 °C for 4 h. The absorbance at 450 nm was measured on a microplate reader. Six independent replicates were conducted for Hepa1-6 and HepG2 cells, respectively.

### Cell death quantification by PI staining

A Propidium Iodide (PI) fluorescent probe (HY-D0815, MCE) was used to measure cell viability. Cells were seeded at a confluency of ~70–80% per well in 6-well plates and incubated overnight. Then, cells were treated according to their respective groups. After the treatment, cells were collected, washed with PBS, and incubated with 1 μg/ml of PI for 1 h. Excessive PI was washed with PBS and Hoechst was used to stain the nuclei. The fluorescence of each group was observed using a fluorescence microscope (Leica Microsystems, Germany) and photographed for subsequent analysis. The percentage of red fluorescent cells was counted by ImageJ. Three independent replicates were conducted for Hepa1-6 and HepG2 cells, respectively, and 200 cells per sample were counted.

### Measurement of lipid peroxidation by C11-BODIPY

The total cellular lipid peroxidation was quantified using a C11-BODIPY (581/591) probe (HY-D1301, MCE). Cells were treated as indicated and harvested. The collected cells were incubated with C11-BODIPY (2 µM) for 30 min at 37 °C in fresh medium. Then, the cells were washed twice with PBS to remove excess C11-BODIPY and were stained with Hoechst for visualization of nuclei. The images were captured using a fluorescence microscope. The intensity of green fluorescence was analyzed by ImageJ. Three independent replicates for Hepa1-6 and HepG2 cells, respectively, and 100 cells per sample were counted.

### Measurement of malondialdehyde (MDA)

Intracellular malondialdehyde (MDA) was measured by MDA assay kit (S0131, Beyotime). Briefly, cells were seeded into 10 cm dishes and cultured overnight. After treatment, cells were harvested and lysed. The protein concentration was quantified by BCA assay. Then, the MDA working solution was added into the total protein of each group and heated at 100 °C for 15 min. After centrifuge, the supernatant was collected, and MDA concentration was measured at 532 nm with a microplate reader. The relative cellular MDA concentration was presented as percentage of control. Three independent replicates were conducted for Hepa1-6 and HepG2 cells, respectively.

### Western blot analysis

Cells were harvested and lysed with protein extraction kit (BB-3121, Bestbio). The concentration of protein extract was quantitated by BCA protein assay reagent (P0010, Beyotime). An equal amount of protein (20 μg) was loaded to SDS-PAGE gels for electrophoresis and then transferred to PVDF membrane. The membrane was washed with TBST and blocked with 2% bovine serum albumin (BSA) for 1 h at room temperature. After that, the membrane was incubated with the primary antibodies against SLC7A11 (DF12509, Affinity Bioscience), GPX4 (DF6701, Affinity Bioscience), Ferritin (DF6278, Affinity Bioscience), LC3 (4108, Cell Signaling Technology), GRP78 (YT5858, Immunoway), ATF4 (11815, Cell Signaling Technology), CHOP (AF6277, Affinity Bioscience), IRE1 (DF7709, Affinity Bioscience), STING (DF12090, Affinity Bioscience), phosphor-STING (AF7416, Affinity Bioscience), Nrf2 (AF0639, Affinity Bioscience), HO-1 (AF5393, Affinity Bioscience), and GAPDH (AF7021). Three independent replicates were conducted for Hepa1-6 and HepG2 cells, respectively. All uncropped Western blot images are provided in Supplementary Material.

### Autophagic flux assay

Autophagic flux assay was used to measure nelfinavir-induced autophagy by quantifying LC3II turnover in the presence or absence of the lysosome inhibitor chloroquine. Compared to nelfinavir alone, a further increase of LC3II in the presence of chloroquine indicates that nelfinavir increases autophagy. In our experiments, nelfinavir (40 μM) treated Hepa1-6 and HepG2 cells were further exposed or not to chloroquine (40 μM) for 2 h before the termination of the experiment. Thereafter, the cells were collected and processed for the detection of LC3II by immunoblot. Three independent replicates were conducted for Hepa1-6 and HepG2 cells, respectively.

### Cycloheximide (CHX) based protein chase assay

Cycloheximide (CHX), a protein synthesis inhibitor, was used to evaluate the stability of GPX4. Hepa1-6 cells were treated CHX (10 μg/mL) together with DMSO (control), nelfinavir (40 μM), or nelfinavir (40 μM) plus chloroquine (40 μM) for 0, 6, 12 and 24 h. Proteins were extracted, and Western blot was performed to detect the expression of GPX4 protein. Three independent replicates were conducted.

### Measurement of glutathione

The measurement of glutathione (GSH) was assessed using GSH and GSSG Assay Kit (S0053, Beyotime) according to the manufacturer’s instructions. Briefly, cells, according to their treatments, were collected and lysed at 4 °C. GSSG in cell lysate was reduced to GSH by glutathione reductase. The formed GSH continuously reduced 2-nitrobenzoic acid (DTNB) to 5-thio-2-nitrobenzoic acid (TNB). Concentration of TNB was measured at 420 nm spectrophotometrically, which reflected the amount of GSH. The results were normalized by protein concentration of each sample. Three independent replicates were conducted for Hepa1-6 and HepG2 cells, respectively.

### Measurement of mitochondrial membrane potential

Mitochondrial membrane potential was measured by staining with JC-1 (MT09, Dojindo). Cells were treated with nelfinavir (40 μM) for 24 h. Subsequently, the cells were collected and incubated in the JC-1 staining solution for 30 min at 37 °C. Hoechst was used to stain the nuclei. After replacing the staining solution with PBS, the cells were observed and photographed under a fluorescence microscope. The red and green fluorescence intensity was analyzed by ImageJ. Three independent replicates for Hepa1-6 and HepG2 cells, respectively, and 100 cells per sample were counted.

### Evaluation of mitochondrial superoxide

MitoSOX red (HY-D1055, MCE) staining was performed for the detection of mitochondrial superoxide production. Hepa1-6 and HepG2 cells were treated with nelfinavir (40 μM) for 24 h. After the treatment, cells were collected and then incubated with MitoSOX red at a concentration of 2 µM for 30 min at 37 °C. Hoechst was used to stain the nuclei. After incubation and washing, fluorescence images were captured using a fluorescence microscope. The fluorescence intensity was analyzed using ImageJ. Three independent replicates were conducted for Hepa1-6 and HepG2 cells, respectively, and 100 cells per sample were counted.

### Detection of Fe^2+^

The intracellular ferrous levels were determined by FerroOrange dye (F374, Dojindo). Hepa1-6 and HepG2 cells were treated with nelfinavir (40 μM) for 24 h. Then, cells were collected and incubated with 1 μM FerroOrange for 30 min at 37 °C. Hoechst was used to stain the nuclei. After incubation and washing, fluorescence images were captured using a fluorescence microscope. The fluorescence intensity was analyzed using ImageJ. Three independent replicates were conducted for Hepa1-6 and HepG2 cells, respectively, and 100 cells per sample were counted.

### Nude mouse xenografts

Male BALB/c nude mice (weighing 16–18 g) aged 6–8 weeks were purchased from Charles River Laboratory. Five million Hepa1-6 cells were suspended with 100 μl PBS and implanted subcutaneously into the right flank of the mice. When the tumor volume reached about 100 mm^3^, mice were randomly divided into three groups (*n* = 4) and administered intraperitoneally (i.p.) with 100 μl of vehicle, nelfinavir (50 mg/kg), or nelfinavir plus sorafenib (50 mg/kg+30 mg/kg) once a day for 10 days. The tumor volume was measured every other day. The mouse experiment was carried out in accordance with the Association for Assessment and Accreditation of Laboratory Animal Care guidelines.

### Statistical analysis

All experiments were conducted at least three times, and the data are presented as the mean ± SD. Statistical significance among different treatments were determined by one-way ANOVA followed by Tukey’s post hoc test. A *p* or adjusted *p* value < 0.05 is considered statistically significant.

## Supplementary information

Supplementary Figures

Full and Uncropped western blots

Fig S2

## Data Availability

Research data are stored in an institutional repository and will be shared upon reasonable request to the corresponding author. Uncropped western blot images are provided in Supplemental Material.
