# Supplementary material for: Nelfinavir triggers ferroptosis by inducing ER stress mediated downregulation of GPX4/GSH system, upregulation of NRF2/HO-1 axis, and mitochondrial impairment in hepatocellular carcinoma cells
Source: Cell Death Discov. 2025 Oct 6;11:444. doi: 10.1038/s41420-025-02761-w (PMC12501057; doi:10.1038/s41420-025-02761-w)
Supplement: Supplementary file 1 — Supplementary Figures [file 41420_2025_2761_MOESM1_ESM.pdf]

Supplementary Figures for

Nelfinavir triggers ferroptosis by inducing ER stress mediated downregulation of GPX4/GSH system, upregulation of NRF2/HO-1 axis, and mitochondrial impairment in hepatocellular carcinoma cells

Running title: Nelfinavir induces ferroptosis via ER stress in HCC

Lei Zhang[1,2*], Xuejun Wang[1,*]

[1]Division of Basic Biomedical Sciences, The University of South Dakota Sanford School of Medicine, Vermillion, South Dakota, USA.

[2]College of Veterinary Medicine, Jilin Agricultural University, Changchun, China

Figure S1

Figure S2

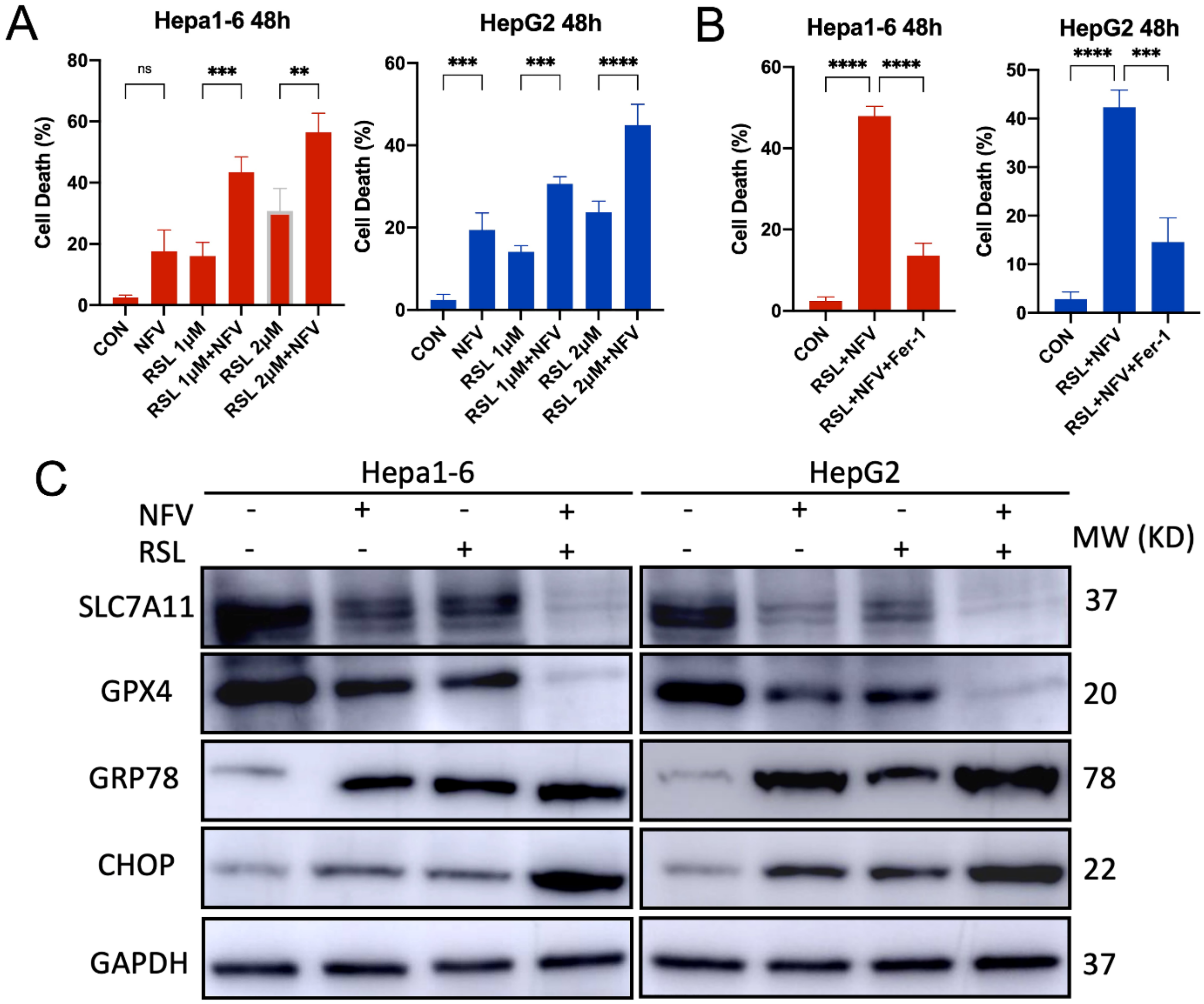

Figure S1. **Nelfinavir potentiates RSL3-induced ferroptosis.** (A) Cell death was measured using CCK-8 assay in Hepa1-6 and HepG2 cells treated with RSL3 (1 μM and 2 μM) with or without nelfinavir (20 μM) at 48 hours. (B) Cell death was measured using CCK-8 assay in Hepa1-6 and HepG2 cells treated with combined nelfinavir (20 μM) and RSL3 (2 μM) with or without Fer-1 (1 μM) at 48 hours. (E) Detection of protein expression of SLC7a11, GPX4, GRP78 and CHOP after 24 hours exposure of sorafenib (10 μM) and/or nelfinavir (20 μM). Data were presented as Mean ± SD. Differences were assessed by one-way ANOVA. $^{**}p < 0.01$, $^{***}p < 0.001$, and $^{****}p < 0.0001$. NFV, nelfinavir. RSL, RSL3.

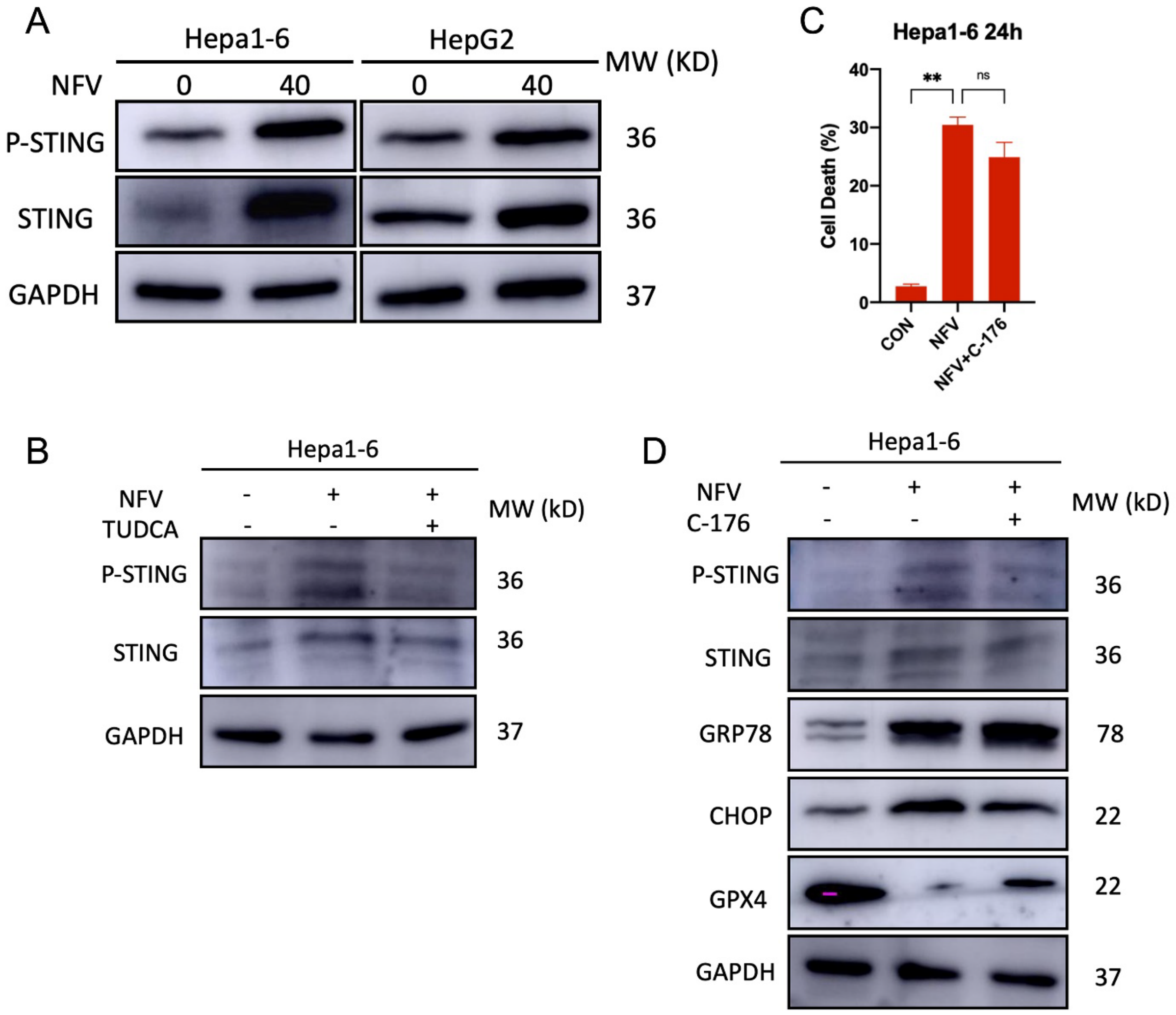

Figure S2. **Nelfinavir (NFV) activates STING.** (**A**) Western blot analyses for phosphorylated STING (P-STING), STING, and GAPDH in Hepa1-6 (n=3) and HepG2 (n=3) cells 24 hours after NFV (40μM) treatment was initiated. (**B**) Effect of TUDCA (200μM) on NFV-induced activation of STING after 24 hours treatment in Hepa1-6 cells (n=3). (**C**) Effect of pre-treatment of Hepa1-6 cells with C-176 (2μM) for 3 hours on NFV-induced cell death after 24 hours treatment. n=3 independent replicates. (**D**) Effect of C-176 on NFV-induced upregulation of ER stress associated protein of GRP78 and CHOP, and downregulation of GPX4 after 24 hours treatment in Hepa1-6 cells (n=3). Data were presented as Mean ± SD. Differences were assessed by one-way ANOVA followed by Tukey's tests. Ns, not significant; ***p*<0.01.
