# Supplementary material for: Nelfinavir triggers ferroptosis by inducing ER stress mediated downregulation of GPX4/GSH system, upregulation of NRF2/HO-1 axis, and mitochondrial impairment in hepatocellular carcinoma cells
Source: Cell Death Discov. 2025 Oct 6;11:444. doi: 10.1038/s41420-025-02761-w (PMC12501057; doi:10.1038/s41420-025-02761-w)

Un-Chopped Western Blot

Figure 2A

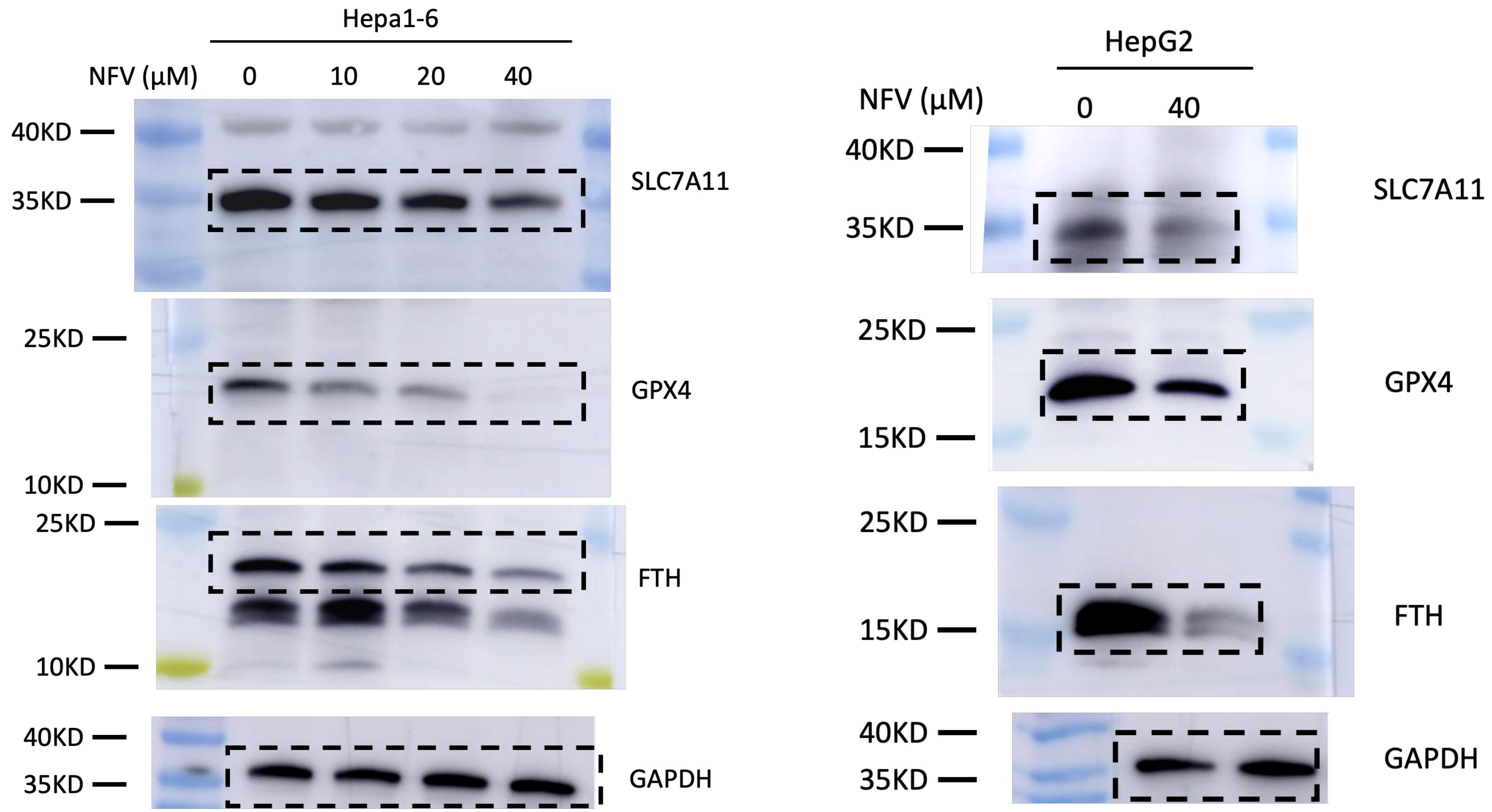

Hepa1-6
NFV (μM) 0 10 20 40
40KD
35KD
SLC7A11
25KD
GPX4
10KD
25KD
FTH
10KD
40KD
35KD
GAPDH
HepG2
NFV (μM) 0 40
40KD
35KD
SLC7A11
25KD
GPX4
15KD
25KD
FTH
15KD
40KD
35KD
GAPDH

Figure 3A

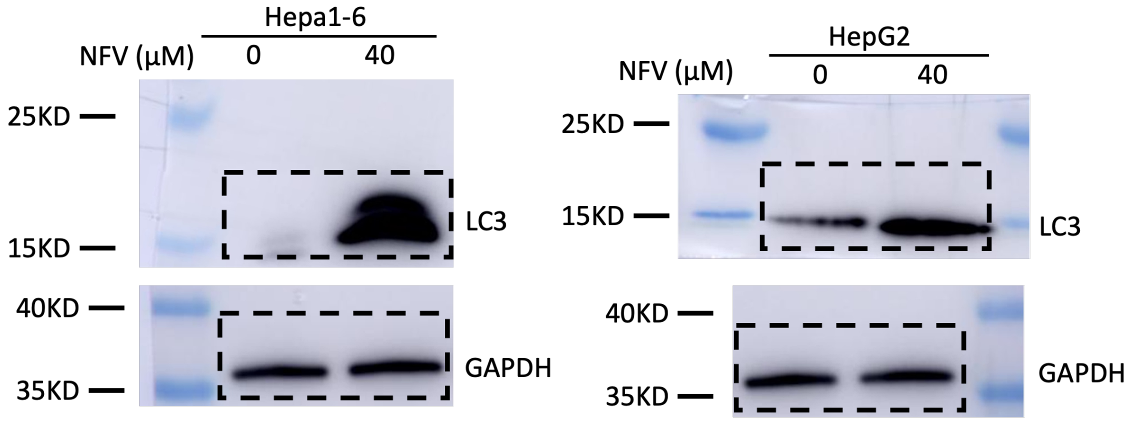

Figure 3B

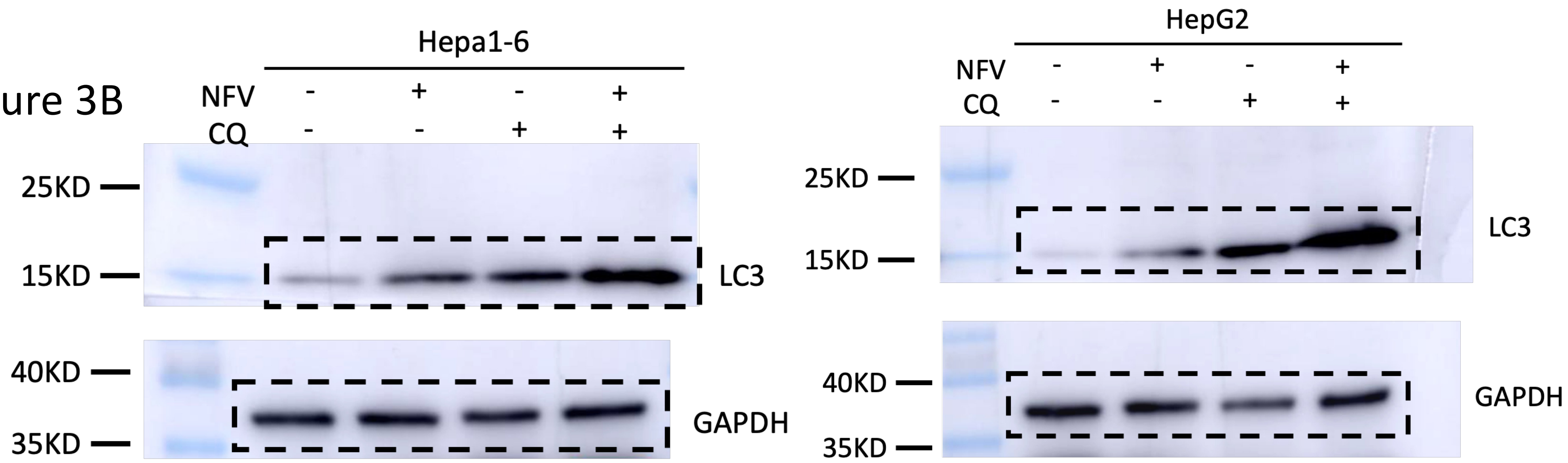

Figure 3C

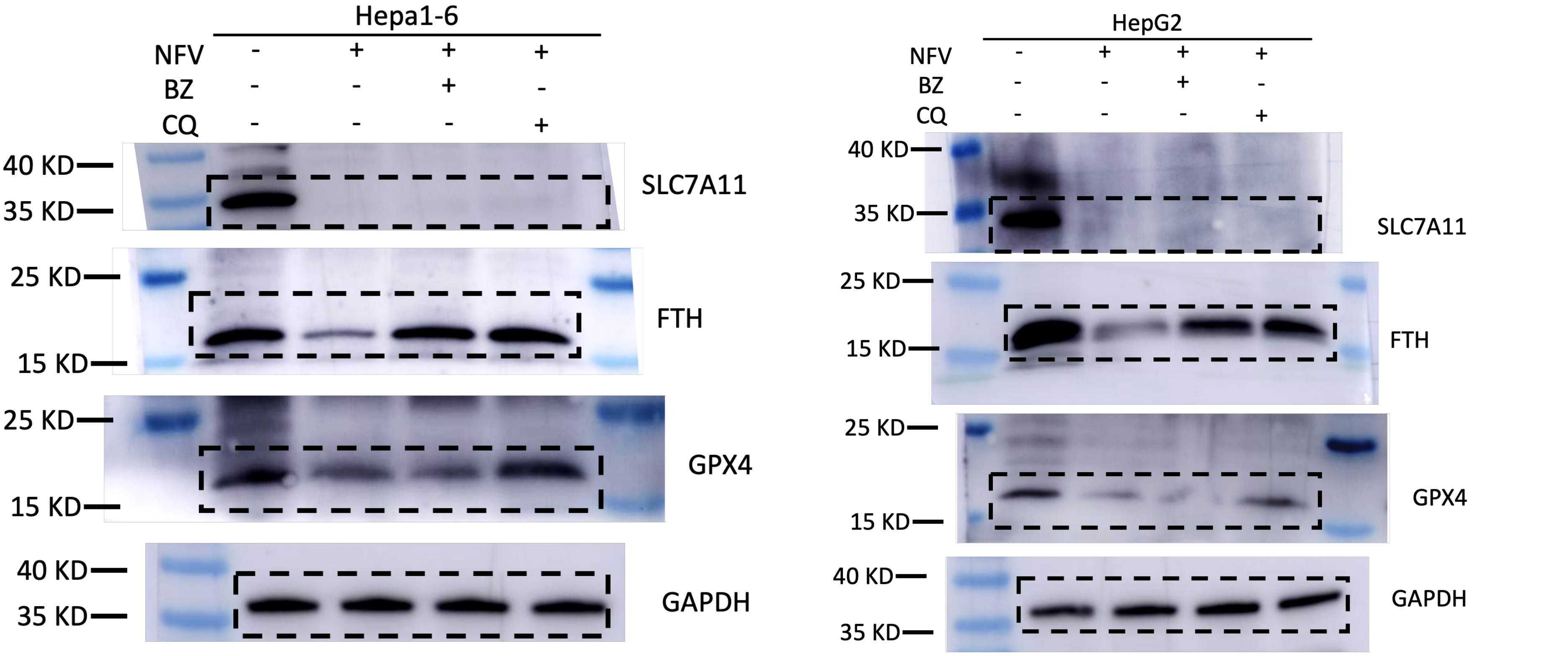
Hepa1-6
NFV - + + +
BZ - - + -
CQ - - - +
40 KD
35 KD
SLC7A11
25 KD
15 KD
FTH
25 KD
15 KD
GPX4
40 KD
35 KD
GAPDH
HepG2
NFV - + + +
BZ - - + -
CQ - - - +
40 KD
35 KD
SLC7A11
25 KD
15 KD
FTH
25 KD
15 KD
GPX4
40 KD
35 KD
GAPDH

Figure 3D

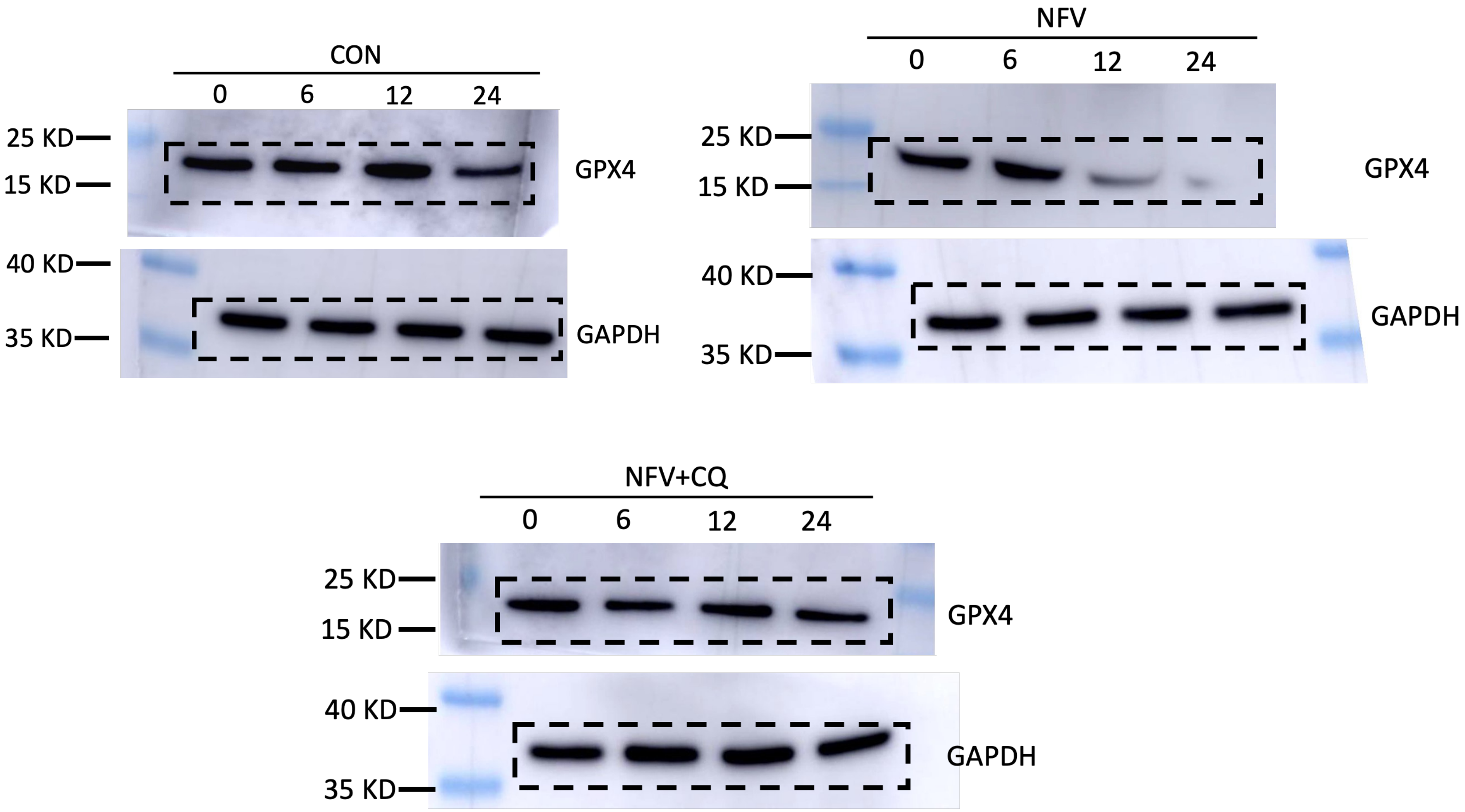
CON
0
6
12
24
25 KD
15 KD
GPX4
40 KD
35 KD
GAPDH
NFV
0
6
12
24
25 KD
15 KD
GPX4
40 KD
35 KD
GAPDH
NFV+CQ
0
6
12
24
25 KD
15 KD
GPX4
40 KD
35 KD
GAPDH

Figure 4A

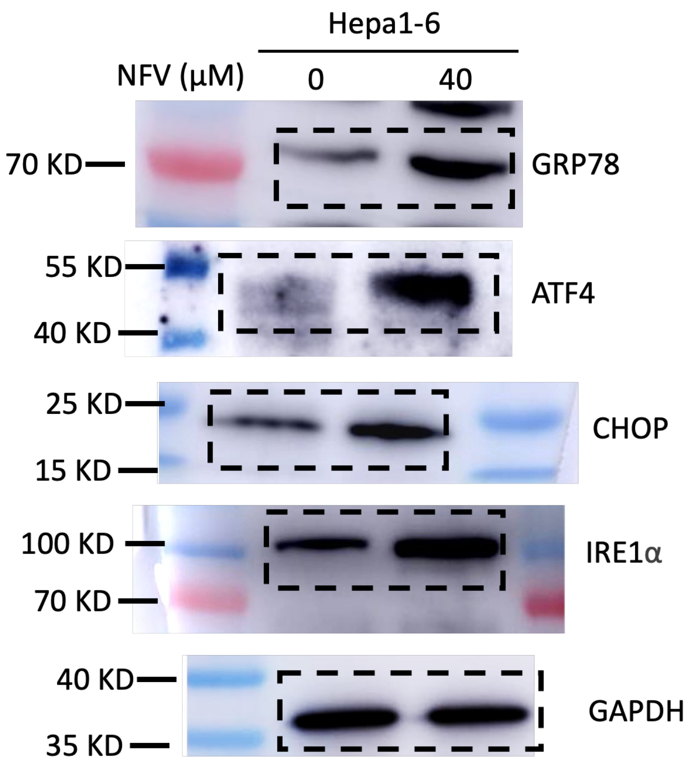

Hepa1-6
NFV (μM)
0
40
70 KD
GRP78
55 KD
40 KD
ATF4
25 KD
15 KD
CHOP
100 KD
70 KD
IRE1α
40 KD
35 KD
GAPDH

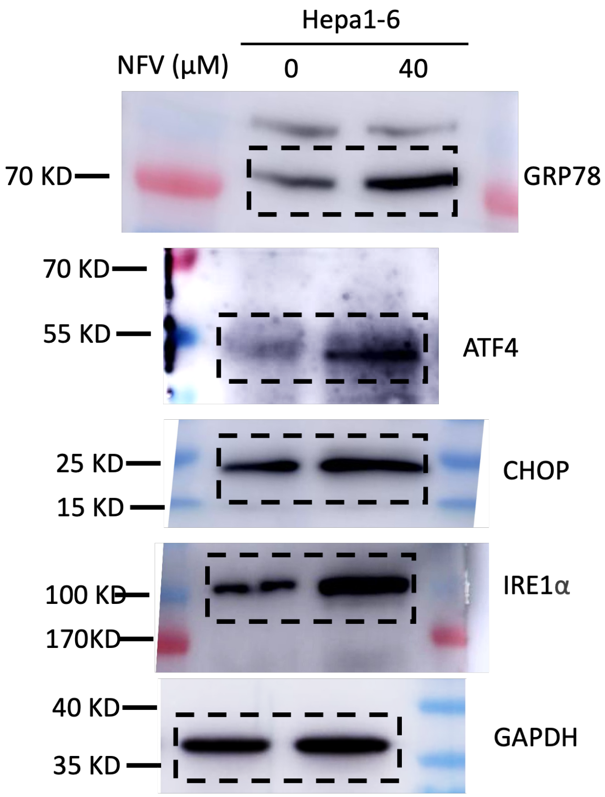

Hepa1-6
NFV (μM)
0
40
70 KD
GRP78
70 KD
55 KD
ATF4
25 KD
15 KD
CHOP
100 KD
170KD
IRE1α
40 KD
35 KD
GAPDH

Figure 4B

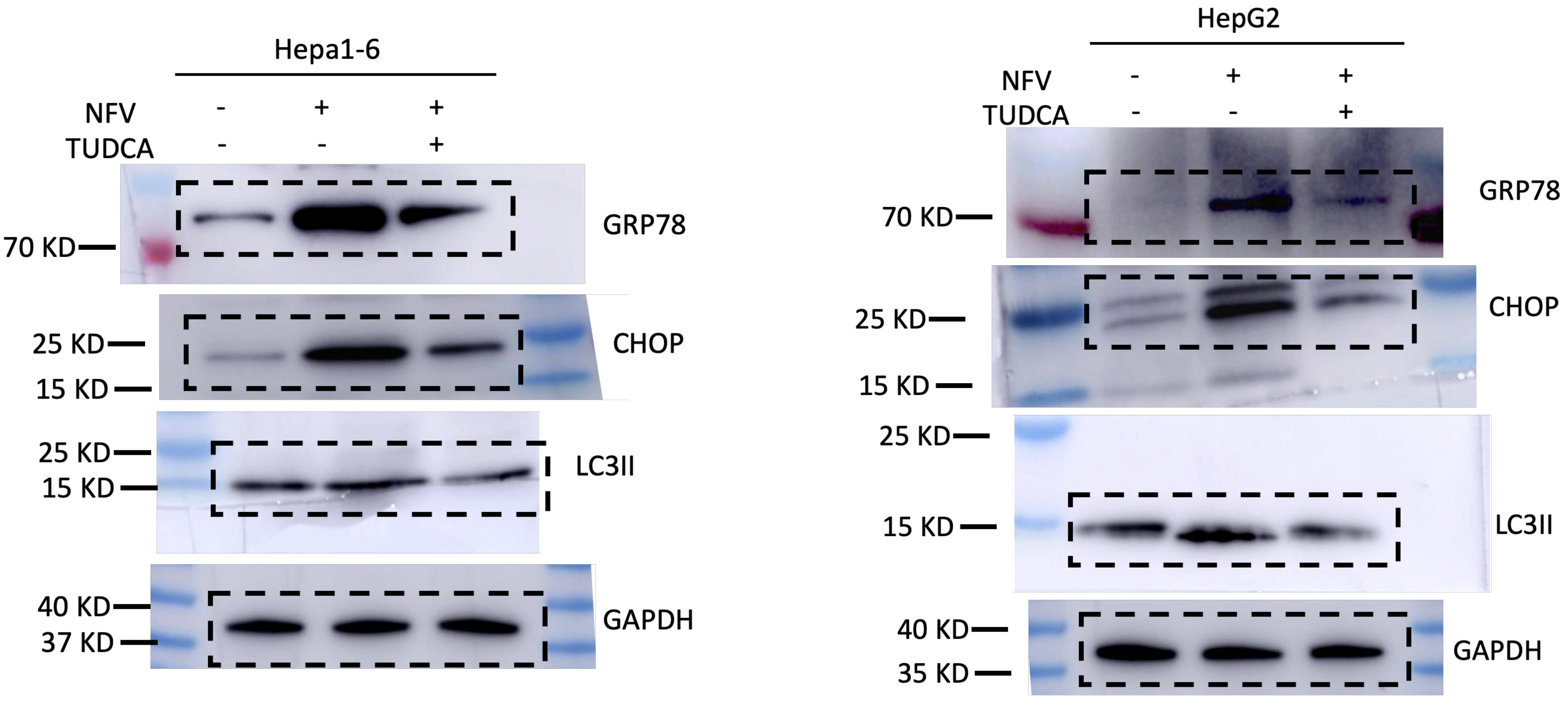

Hepa1-6
NFV - + +
TUDCA - - +
70 KD
GRP78
25 KD
15 KD
CHOP
25 KD
15 KD
LC3II
40 KD
37 KD
GAPDH
HepG2
NFV - + +
TUDCA - - +
70 KD
GRP78
25 KD
15 KD
CHOP
25 KD
15 KD
LC3II
40 KD
35 KD
GAPDH

Figure 4G

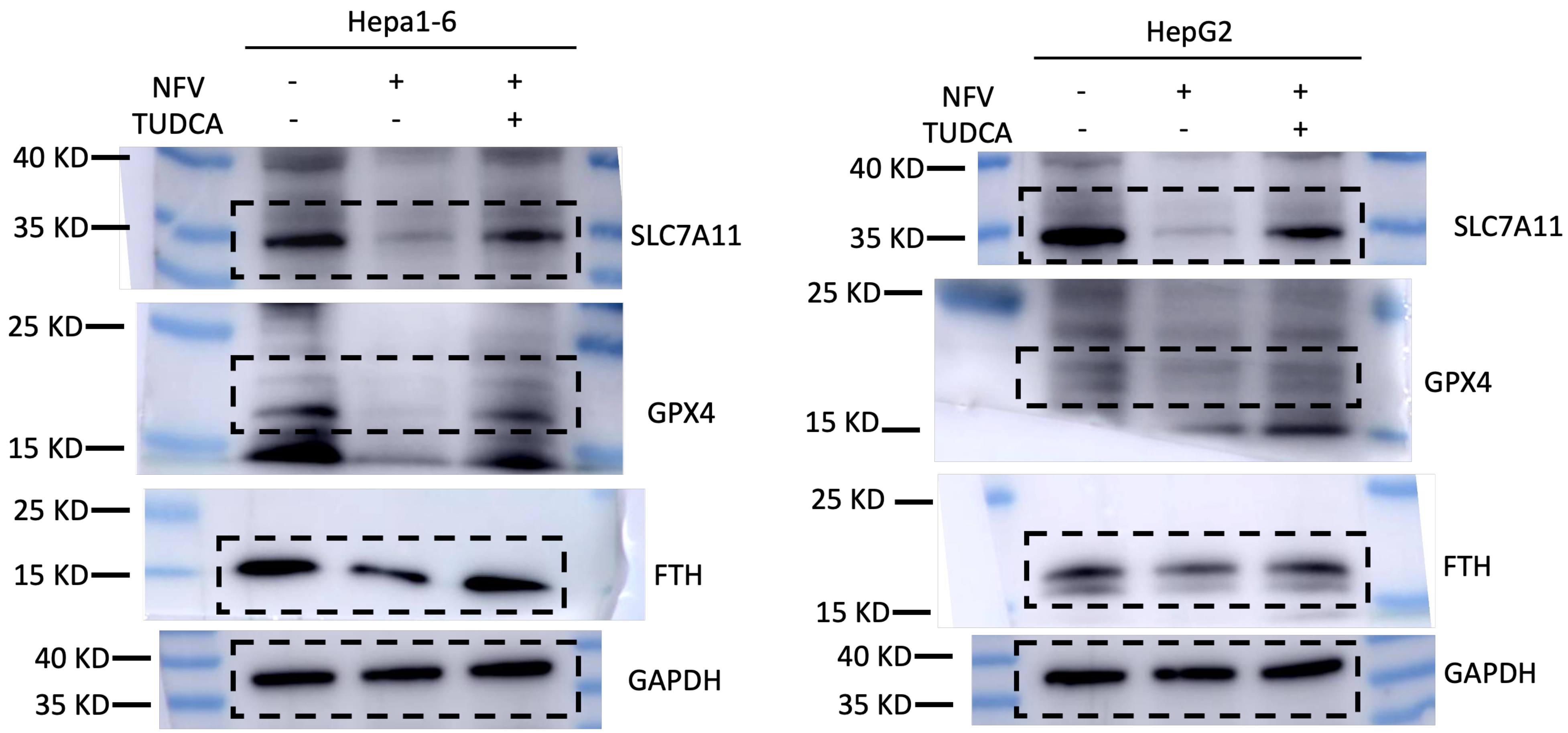

Hepa1-6
NFV - + +
TUDCA - - +
40 KD
35 KD
SLC7A11
25 KD
15 KD
GPX4
25 KD
15 KD
FTH
40 KD
35 KD
GAPDH
HepG2
NFV - + +
TUDCA - - +
40 KD
35 KD
SLC7A11
25 KD
15 KD
GPX4
25 KD
15 KD
FTH
40 KD
35 KD
GAPDH

Figure 5A

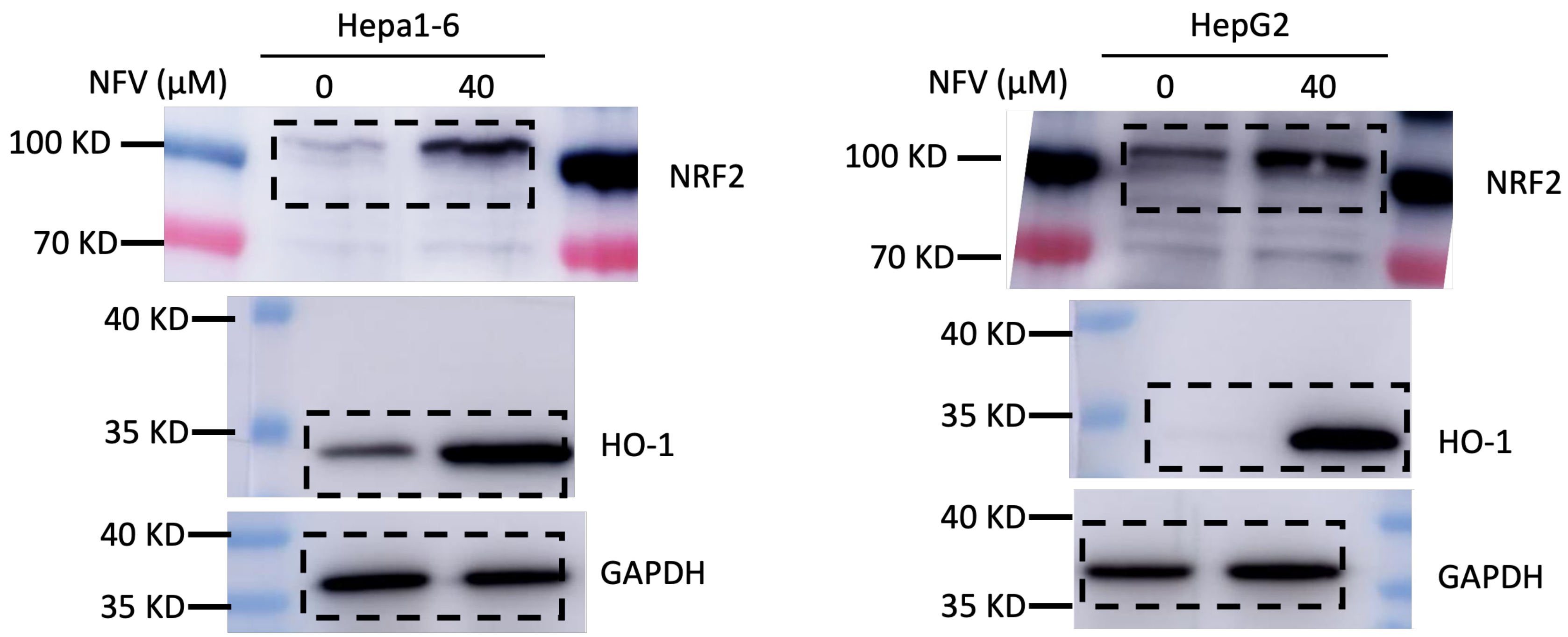
Hepa1-6
NFV (μM)
0
40
100 KD
70 KD
NRF2
40 KD
35 KD
HO-1
40 KD
35 KD
GAPDH
HepG2
NFV (μM)
0
40
100 KD
70 KD
NRF2
40 KD
35 KD
HO-1
40 KD
35 KD
GAPDH

Figure 5B

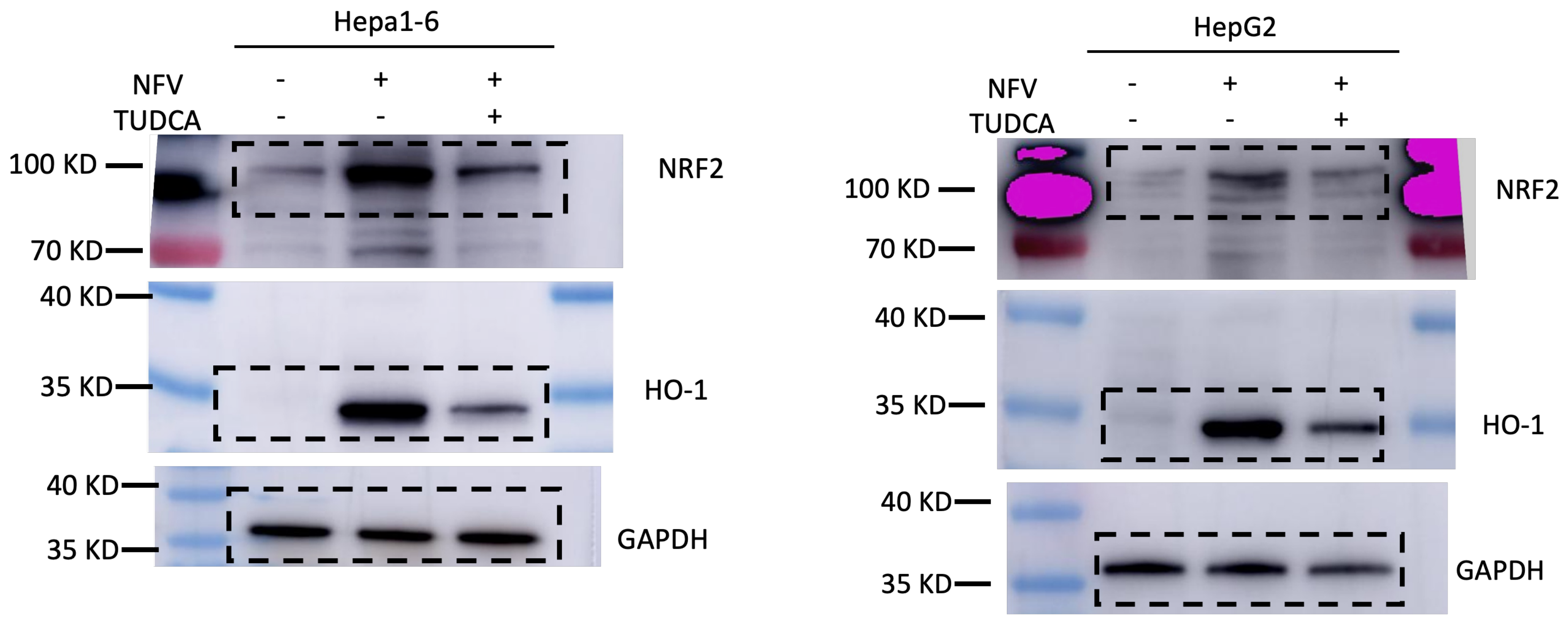
Hepa1-6
NFV - + +
TUDCA - - +
100 KD
70 KD
NRF2
40 KD
35 KD
HO-1
40 KD
35 KD
GAPDH
HepG2
NFV - + +
TUDCA - - +
100 KD
70 KD
NRF2
40 KD
35 KD
HO-1
40 KD
35 KD
GAPDH

Figure 7E

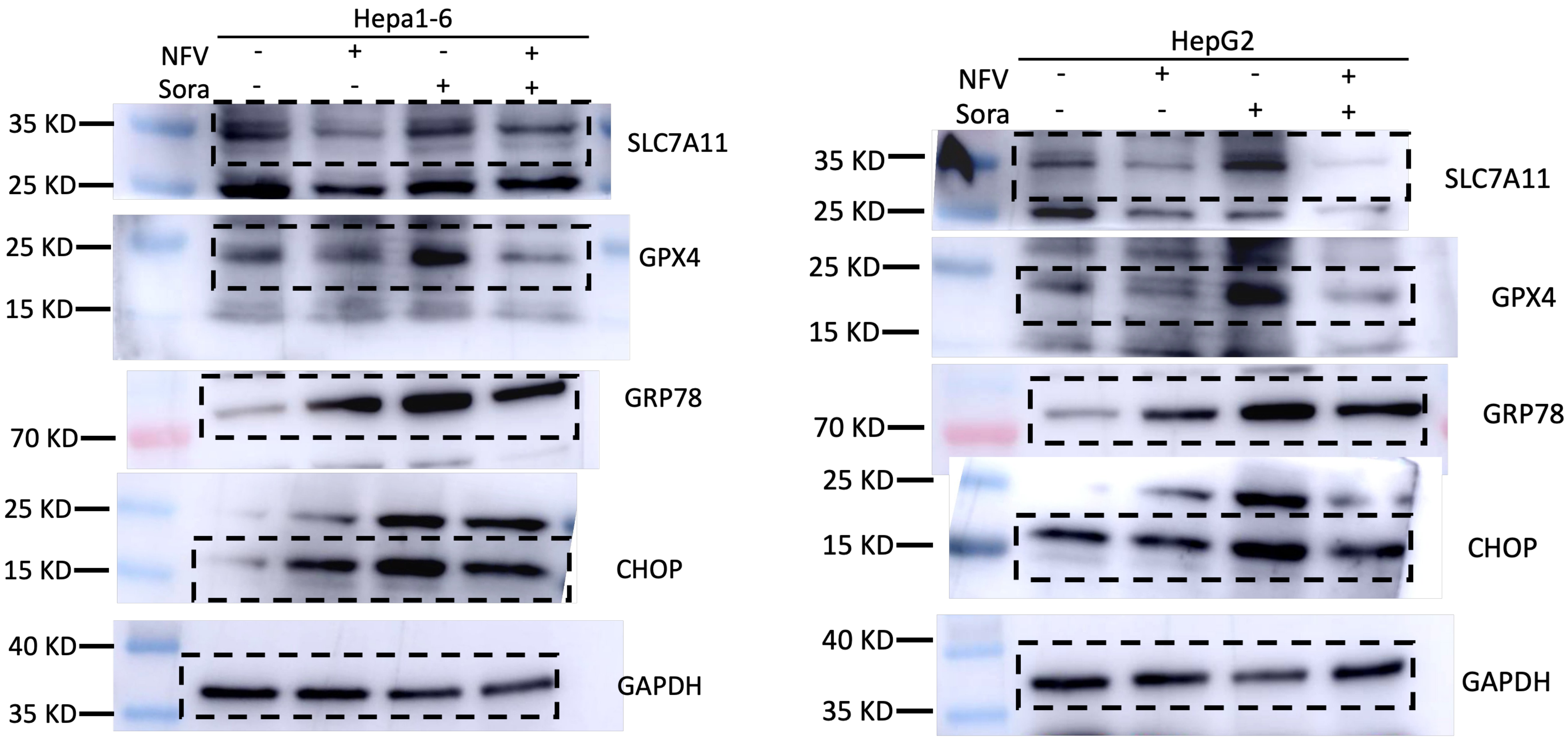
Hepa1-6
NFV - + - +
Sora - - + +
35 KD
25 KD
SLC7A11
25 KD
15 KD
GPX4
GRP78
70 KD
25 KD
15 KD
CHOP
40 KD
35 KD
GAPDH
HepG2
NFV - + - +
Sora - - + +
35 KD
25 KD
SLC7A11
25 KD
15 KD
GPX4
70 KD
GRP78
25 KD
15 KD
CHOP
40 KD
35 KD
GAPDH

Figure S1C

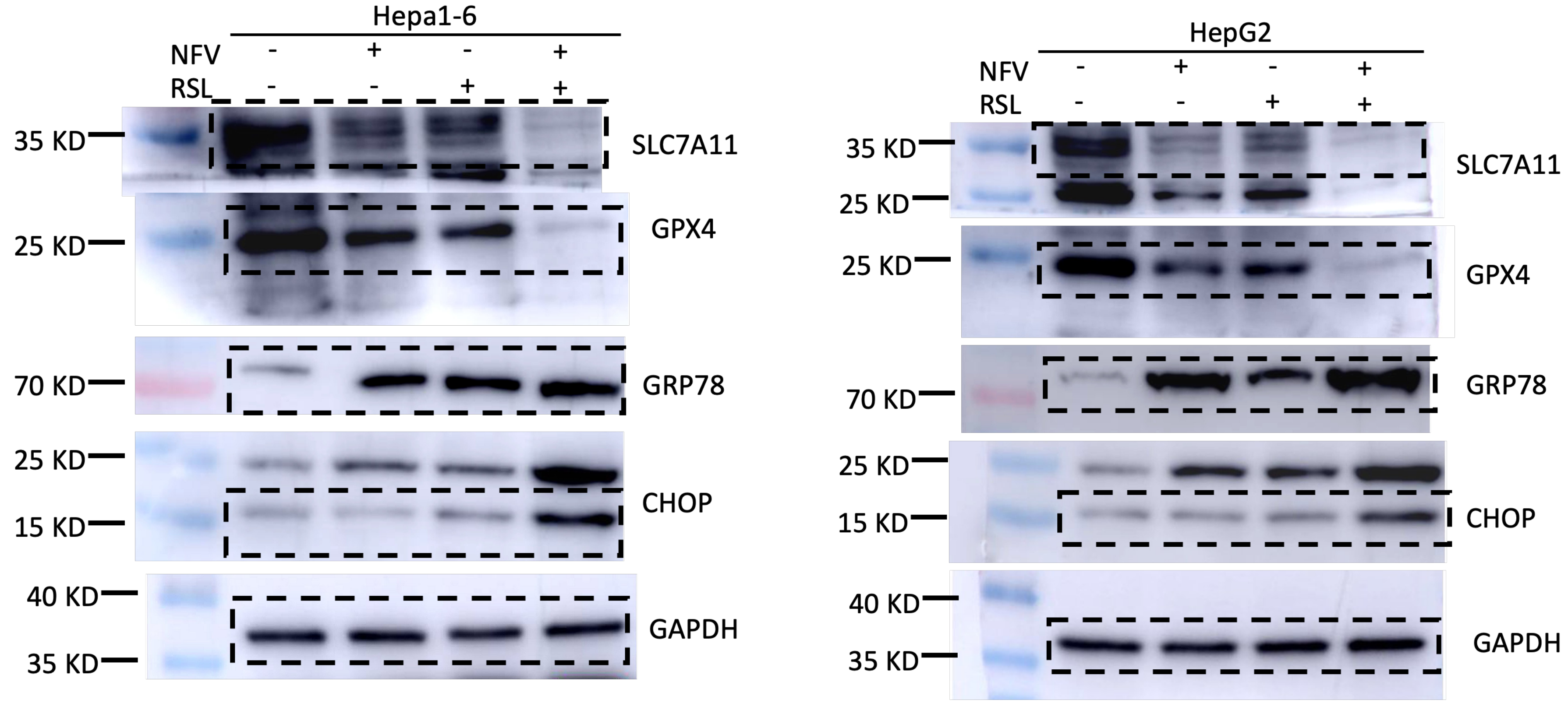
Hepa1-6
NFV - + - +
RSL - - + +
35 KD
SLC7A11
25 KD
GPX4
70 KD
GRP78
25 KD
15 KD
CHOP
40 KD
35 KD
GAPDH
HepG2
NFV - + - +
RSL - - + +
35 KD
25 KD
SLC7A11
25 KD
GPX4
70 KD
GRP78
25 KD
15 KD
CHOP
40 KD
35 KD
GAPDH

Figure S2A

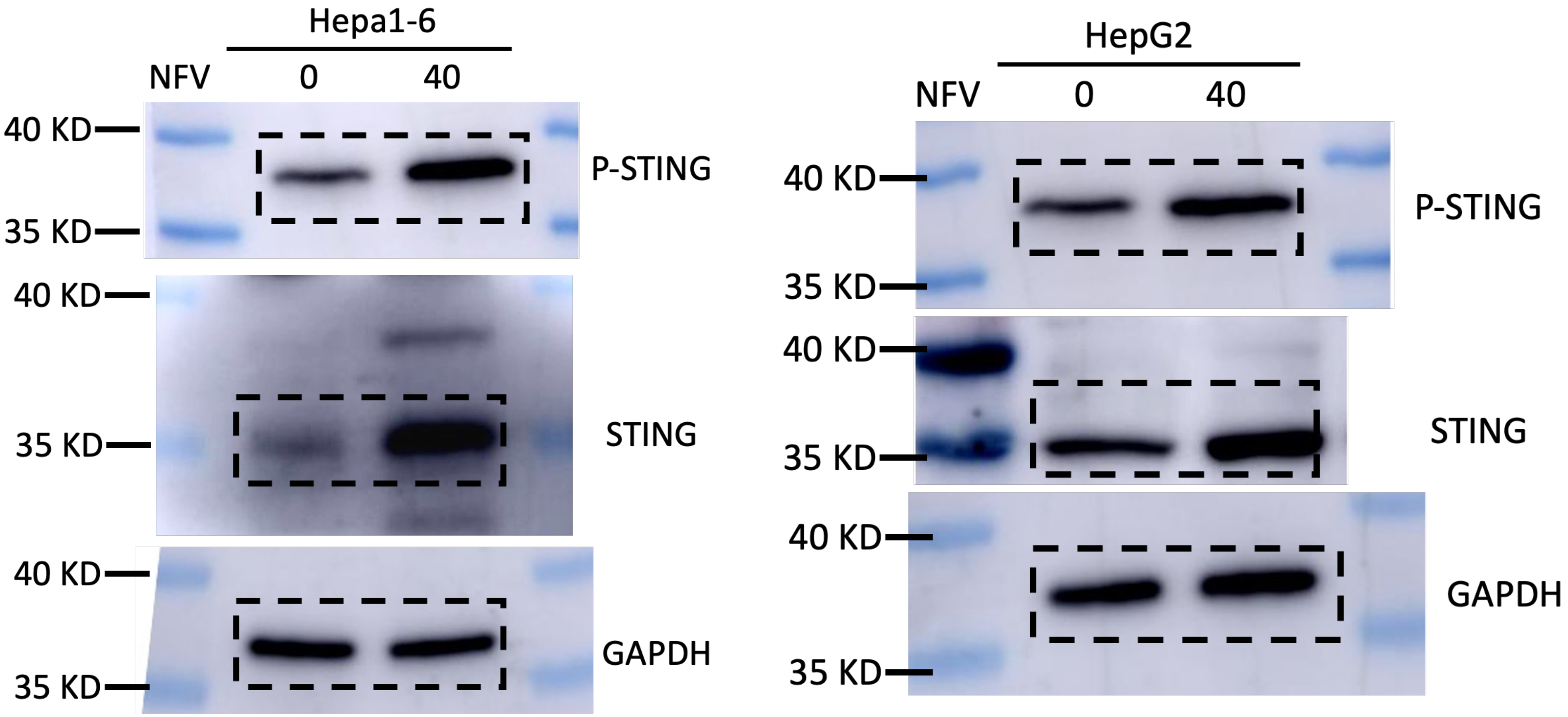

Hepa1-6
NFV
0
40
40 KD
35 KD
P-STING
40 KD
35 KD
STING
40 KD
35 KD
GAPDH
HepG2
NFV
0
40
40 KD
35 KD
P-STING
40 KD
35 KD
STING
40 KD
35 KD
GAPDH

Figure S2B

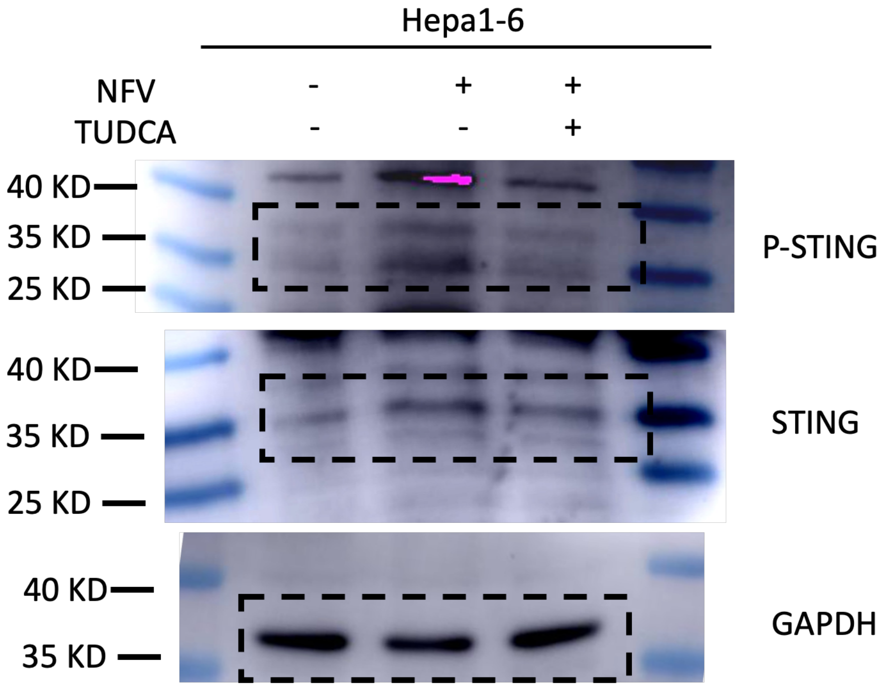

Figure S2D

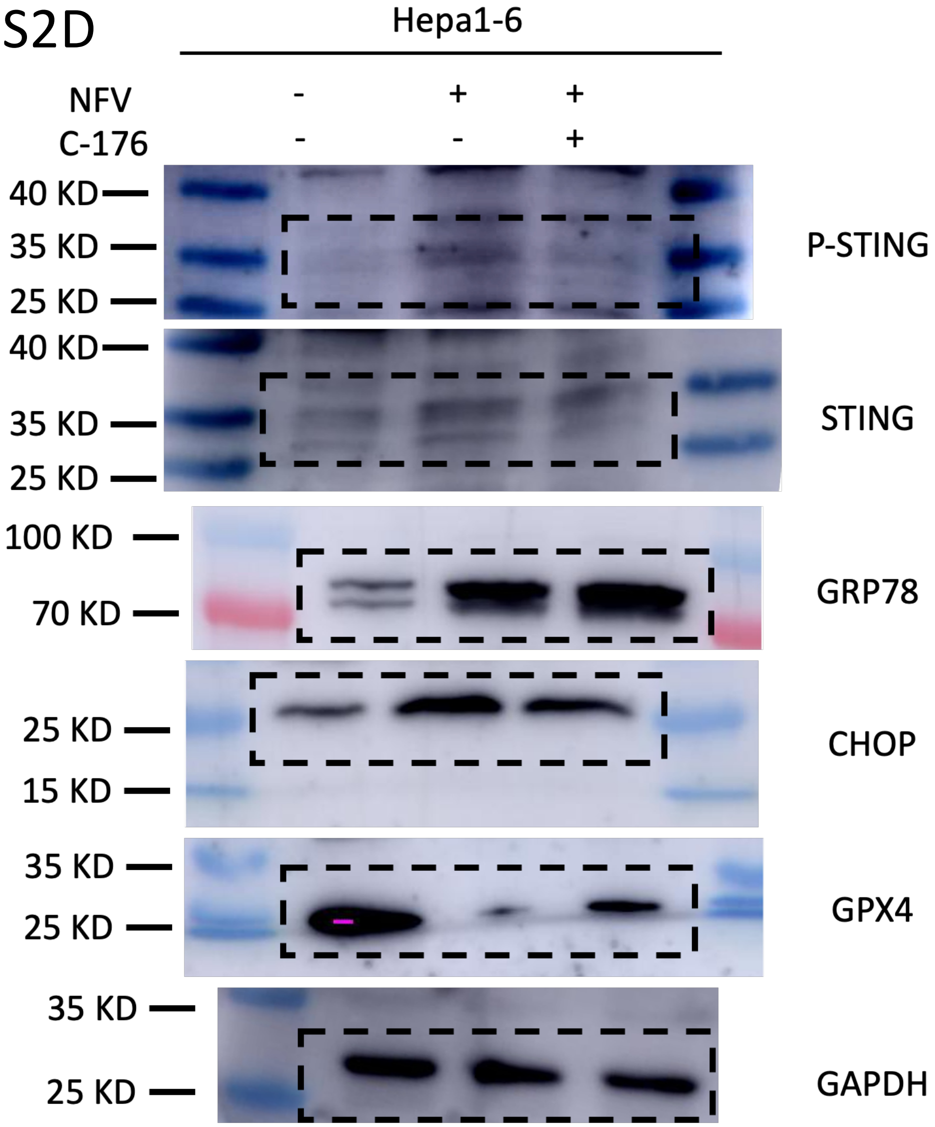

Supplement: Supplementary file 2 — Full and Uncropped western blots [file 41420_2025_2761_MOESM2_ESM.pdf]
