# Supplementary figures and images for: Nelfinavir triggers ferroptosis by inducing ER stress mediated downregulation of GPX4/GSH system, upregulation of NRF2/HO-1 axis, and mitochondrial impairment in hepatocellular carcinoma cells
Source: Cell Death Discov. 2025 Oct 6;11:444. doi: 10.1038/s41420-025-02761-w (PMC12501057; doi:10.1038/s41420-025-02761-w)

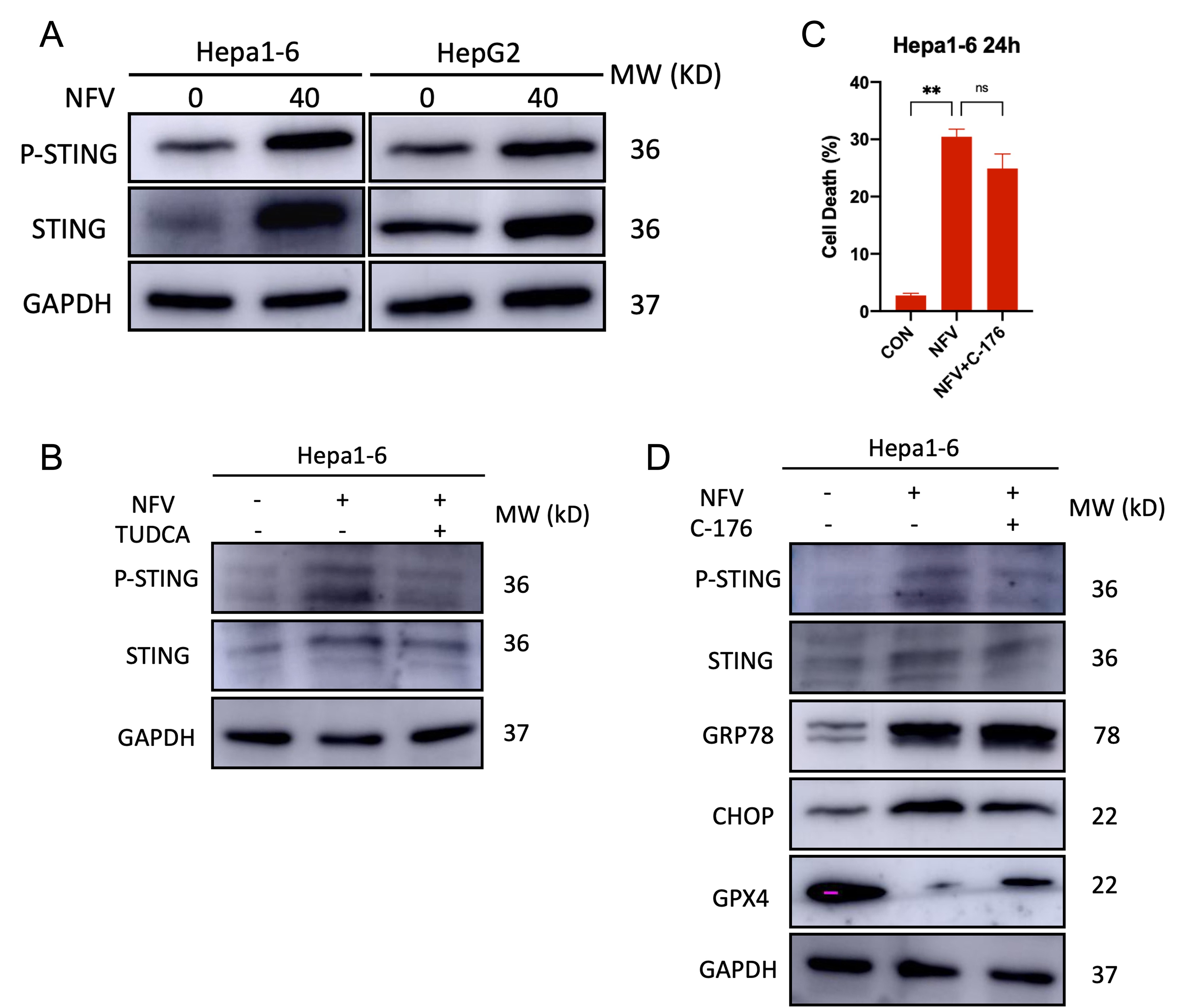

Supplement: Supplementary file 3 — Fig S2 [file 41420_2025_2761_MOESM3_ESM.tif]
